# Interpersonal Synchrony During a Client–Therapist Role‐Play Interaction: The Role of Modalities, Facets, and Relational Context

**DOI:** 10.1111/psyp.70436

**Published:** 2026-11-01

**Authors:** Noha M. Yassen, Jacobien M. van Peer, Ger P. J. Keijsers, Leentje Vervoort, Travis J. Wiltshire

**Affiliations:** ^1^ Behavioural Science Institute Radboud University Nijmegen the Netherlands; ^2^ Department of Computational Cognitive Science Tilburg University Tilburg the Netherlands

**Keywords:** client–therapist interaction, interpersonal synchrony, multimodal synchrony, physiological synchrony, relational context, working alliance

## Abstract

Interpersonal synchrony (IS), the temporal coordination between individuals during interaction, is a complex phenomenon that manifests across different modalities (e.g., behavioral and physiological) and coordination facets (e.g., strength and flexibility). In psychotherapy, IS is increasingly considered a potential embodied marker of the client–therapist relationship. However, little is known about how relational context shapes IS coordination patterns, or how they relate to perceived relationship quality. This study aimed to identify IS coordination patterns associated with two relational contexts (emotion‐focused vs. solution‐focused conversations) and to examine how these patterns relate to subjective perceptions of the client–therapist relationship. 168 psychology students (84 dyads) participated in two consecutive 13‐min client–therapist role‐play conversations with different goals: exploring emotions (emotion‐focused) and brainstorming solutions (solution‐focused). Windowed Cross‐Lagged Correlation was used to quantify IS from upper‐body movement (using Motion Energy Analysis), high‐frequency heart rate variability, and electrodermal activity (average and slope). For each modality, six IS facets were extracted, representing three facet families: strength, flexibility, and leading–following dynamics. IS was above chance level across all modalities and facets in both conditions (*d*s ≈0.26–1.09). Although the context manipulation was effective at a subjective level, no condition differences in IS were observed after correction for multiple testing. Analyses accounting for dyads' interaction history (i.e., sequence of conditions) revealed context‐dependent effects in two modalities: movement strength and EDA_Slope_ leading–following dominance. Both modalities were also related with the perceived emotional bond, but not with perceived collaboration, and these associations varied as a function of relational context. These findings suggest that contextual factors, including conversation goals and dyadic history shape coordination dynamics. Implications for modality and facet selection, and context manipulation in IS research are discussed.

## Impact Statement

Our findings show that interpersonal synchrony (IS) is both multimodal and multifaceted, with context effects depending on specific modality and facet combinations. Critically, dyadic history (i.e., condition sequence) moderated IS dynamics, highlighting the need to refine how context is conceptualized in IS research. Additionally, IS was selectively linked to perceived emotional bond, but not perceived collaboration, supporting a link between IS and social relational processes.

## 1 Introduction

Interpersonal synchrony (IS), the moment‐to‐moment temporal coordination between individuals during social interaction, has received increasing attention across a range of research fields. Examples include developmental psychology (Birk et al. 2022; Feldman 2007), research on romantic partners (Coutinho et al. 2019; Timmons et al. 2015), joint action research (van der Wel et al. 2021), relational neuroscience (De Felice et al. 2025; Levy et al. 2021), teamwork (Mayo et al. 2021; Palumbo et al. 2017; Wiltshire et al. 2019), and psychotherapy (Kleinbub 2017; Nyman‐salonen and Kykyri 2022; Ramseyer and Tschacher 2011; Wiltshire et al. 2020). Despite the large body of work linking IS to prosocial and relational processes (for reviews, e.g., daSilva and Wood 2024; Hu et al. 2022), understanding its underlying mechanisms and its relation to perceived relationship quality remains challenging. One reason for this is that IS is inherently a multimodal and multifaceted emergent phenomenon (Kelso 2022; Nowak et al. 2017; Vial and Cornejo 2022). During social interactions, individuals coordinate across multiple bodily *modalities* (e.g., movement and physiology), and the way this coordination unfolds over time manifests in different forms or dynamic *facets* (e.g., interaction partners may move in and out of IS or alternate in leading and following roles). A further challenge lies in the context‐dependent nature of IS (Gordon et al. 2024; Gordon and Bartsch 2026; Scheidt et al. 2021). As an emergent phenomenon, IS patterns observed at any given moment are shaped by the *context* (e.g., goals and histories of the interaction partners), which constrains and organizes social interactions by defining what is required and what is possible in that moment (Juarrero 2023). The role of modalities, facets, and context in understanding IS, is particularly evident in psychotherapy research, where IS has been extensively studied as a potential marker of the perceived quality of the client–therapist relationship or working alliance (WA; Atzil‐slonim et al. 2023; Koole and Tschacher 2016; Wiltshire et al. 2020). The WA consists of an emotional bond component and a collaborative component involving agreement on treatment goals and tasks (Wampold and Flückiger 2023) and is consistently found to be a strong predictor of treatment outcomes (Flückiger et al. 2018; Horvath et al. 2011). WA has most commonly been assessed in relation to IS using the Working Alliance Inventory (WAI), or one of its validated short forms (e.g., Cohen et al. 2021; Reich et al. 2014; Tal et al. 2022; Bar‐Kalifa et al. 2019), although some studies have used alternative methods such as the Bern Post Session Report (BPSR) (e.g., Ramseyer and Tschacher 2011, 2014). Despite being heavily studied, the observed relation between IS and WA remains inconsistent: Some studies report positive associations (Cohen et al. 2021; Ramseyer and Tschacher 2011), others negative (Reich et al. 2014), and some no relation at all (Ramseyer 2020a). In the following subsections, we argue that accounting for modalities, facets, and context could help explain these inconsistencies in IS research.

### 1.1 Modalities of Interpersonal Synchrony

Social interaction can be conceptualized as a dynamic complex system in which not only individuals interact but also multiple bodily modalities within and between individuals continuously interact and coordinate (Gelo and Salvatore 2016; Mayo and Gordon 2020). Movement IS, or the coordination of nonverbal behaviors between individuals, has been the most extensively studied modality, largely due to advances in motion‐tracking technologies, which enable accessible and noninvasive quantification of body movement from video recordings (Fujiwara and Yokomitsu 2021; Kleinbub and Ramseyer 2021; Ramseyer 2020b). Despite many studies, the functional meaning of movement IS remains not fully understood. Early work on nonverbal mirroring and behavioral matching has linked movement IS to embodied rapport and social affiliation (Hove and Risen 2009; Vacharkulksemsuk and Fredrickson 2012). In psychotherapy research, IS of different body parts has been linked to different therapeutic outcomes: IS in upper‐body movements has been shown to predict session‐level therapeutic success and WA ratings (BPSR) in an outpatient sample primarily comprising patients with anxiety and affective disorders, whereas head movement IS predicted longer term treatment outcomes (Ramseyer and Tschacher 2014).

Similarly, physiological IS, or the temporal alignment of physiological processes between individuals, has received increasing attention in the study of natural social interactions due to advances in wearable technologies. Physiological IS is commonly conceptualized in terms of coordination of the autonomic nervous system (ANS), with a distinction between modalities reflecting sympathetic nervous system (SNS) activity and those reflecting parasympathetic nervous system (PNS) activity (Denk et al. 2024). The SNS is primarily associated with increased physiological arousal, whereas the PNS is more associated with regulation (Shaffer et al. 2014). In psychophysiological research, electrodermal activity (EDA) is a well‐established marker of SNS activity (Boucsein et al. 2012; Posada‐Quintero and Chon 2020), while high‐frequency heart rate variability (HF‐HRV) is commonly used as an index of PNS activity (Shaffer et al. 2014; Shaffer and Ginsberg 2017). Importantly, emerging evidence suggests that IS in these different branches of the ANS may relate differently to interpersonal processes. For example, a recent review found that IS in SNS‐related measures (including EDA) was generally positively associated with relationship outcomes, whereas IS in PNS‐related measures (including HF‐HRV) showed negative associations with such outcomes (Mayo et al. 2021).

Although multimodal approaches are essential for capturing the full spectrum of embodied relational processes underlying the working alliance (Nyman‐salonen and Kykyri 2022), and social interaction more broadly, psychotherapy research shows a fragmented body of findings (Kleinbub 2017). For example, EDA IS between client and therapist has been linked to the emotional bond component of the working alliance (WAI), but not to its collaborative component, in clients with test anxiety (Bar‐Kalifa et al. 2019). One multimodal study further suggested that the relationship between movement IS and the WA (WAI) may be moderated by heart rate IS in a depressed client (Tal et al. 2022). Another multimodal study suggested EDA IS and movement IS to reflect distinct processes during couple therapy (Kykyri et al. 2024). Although these findings suggest that different modalities may index distinct relational processes, in the absence of a unifying theoretical framework (Kleinbub 2017), it remains difficult to synthesize meaningful conclusions about what different IS modalities represent or how they relate to client–therapist relational processes, particularly since all existing studies are observational in nature (Atzil‐slonim et al. 2023; Wiltshire et al. 2020), which limits causal inference.

### 1.2 Facets of Interpersonal Synchrony

An additional layer of complexity arises from the fact that even within a single modality, multiple facets or patterns of coordination can be observed. Most of the above‐mentioned psychotherapy studies have operationalized IS focusing on synchrony *strength*, the overall degree to which interaction partners' signals are aligned across a given episode, often the duration of an entire therapy session. However, methodological developments, particularly the use of windowed cross‐lagged correlation (WCLC) and peak‐picking algorithms, suggest that IS comprises multiple facets of coordination dynamics that may reflect different underlying functions (Moulder et al. 2018; Schoenherr et al. 2019). As a result, other facets have begun to receive increasing attention. One such facet is *flexibility*, referring to the extent to which interaction partners move in and out of synchrony over time (Gordon et al. 2024). Another interesting facet concerns *leading–following* dynamics, which captures who leads and who follows at a given moment in the interaction.

Taken together, WCLC allows for the distinction between at least three families of IS facets, *strength*, *flexibility*, and *leading–following* dynamics (Moulder et al. 2018), each of which may serve different functional roles within an interaction. Within each of these facet families, different indices can be extracted depending on how the time‐series data are analyzed. For instance, peak‐detection algorithms can be applied to identify moments of maximal and stable coordination (Boker et al. 2002; Moulder et al. 2018). Accordingly, some studies report peak‐based indices (e.g., peak strength), whereas others rely on *global* indices, such as the average strength of IS across all time points (Atzil‐slonim et al. 2023). A similar distinction applies to *flexibility*, which can be quantified either as a *global* measure (e.g., variability in IS over the whole‐time window; Gordon et al. 2024), or in terms of variability during *peak* moments (Moulder et al. 2018). In contrast, research on leading–following dynamics has primarily focused on directional *dominance*, or who tends to lead the interaction overall. However, an additional largely unexplored facet is the degree of *switching* between leading and following roles (Moulder et al. 2018), which may be particularly relevant in contexts where adaptive flexibility plays a central role.

### 1.3 Relational Context Effects on Interpersonal Synchrony

Another pivotal step in understanding the role IS serves in relationships is to conceptualize it as an emergent phenomenon that is shaped within a relational context (Nowak et al. 2017). Multiple theoretical contributions have highlighted the importance of context when studying IS (Gordon et al. 2024; Gordon and Bartsch 2026; Scheidt et al. 2021). From a complex systems perspective, Juarrero (2023) conceptualizes context as a dynamic web of constraints, in which multiple factors such as goals, histories, and situational conditions interact to shape which actions and interactions are possible in each moment. Within this framework, relational goals (e.g., collaborating to solve a problem) can be understood as higher level, context‐dependent constraints that organize how individuals coordinate with one another. These constraints do not dictate specific behaviors, but rather shape the space of possible coordination patterns, allowing certain forms of IS to emerge while constraining others (Juarrero 2023). This perspective aligns with the idea that changing task demands (e.g., goals) can give rise to different configurations of coordination dynamics (daSilva and Wood 2024; Fusaroli et al. 2014; Gordon et al. 2024). In other words, different relational goals may push dyads into different coordination configurations (i.e., affecting different facets or modalities) depending on what the dyads want to achieve.

Few psychotherapy studies investigated how patterns of IS differ across therapeutic contexts. For instance, in clients with test anxiety, the association between EDA IS and treatment outcomes appears to depend on the therapeutic context. Higher EDA IS was associated with higher client wellbeing during imagery rescripting segments, but not during cognitive‐behavioral segments (Prinz et al. 2022). In a later multimodal study using the same treatment protocol, lower EDA IS has been associated with greater symptom improvement during cognitive interventions, but not during emotion‐focused interventions, whereas the relationship between movement IS and symptom change did not differ between intervention types (Uhl et al. 2025). A group couple therapy study found EDA IS in therapist–therapist dyads but not in client–client dyads, while respiration IS did not show this pattern (Coutinho et al. 2025), suggesting that IS patterns are structured by interactional roles. The role of context is further illustrated in experimental studies from other domains showing, for example, that physiological IS varies systematically between competitive and cooperative tasks (Chanel et al. 2012; Yarmolovsky and Geva 2024). Taken together, these findings suggest that IS is not a uniform process and varies as a function of context‐specific constraints. However, studies investigating the effects of context on multimodal IS and relationship quality are scarce. Furthermore, because psychotherapy studies are primarily observational, they are limited in their ability to draw causal inferences about the role of context in shaping coordination dynamics, which likely contributes to inconsistent findings regarding the relationship between WA and IS. It remains difficult to integrate findings from multiple IS modalities, facets, and contextual influences within a unified experimental and theoretical framework. To address this gap, we designed a novel client–therapist role‐play interaction task in which we measured multimodal IS and WA while we experimentally manipulated the relational context.

### 1.4 This Study

In this study, we aimed to create relational contexts that map onto the core components of the WA (Wampold and Flückiger 2023): an *emotional* component (i.e., the emotional bond between client and therapist) and a *collaborative* component (i.e., collaboration and agreement on therapeutic goals and tasks). Accordingly, we designed two relational contexts in which a “client” and “therapist” discussed the client's problem, with distinct goals: an emotion‐focused context (goal: exploring emotions) and a solution‐focused context (goal: brainstorming about solutions). Video and physiology were recorded throughout both interactions to compute movement, EDA and HF‐HRV as well as the IS facets strength, flexibility, and leading‐following dynamics. To ensure the effectiveness of our manipulation, we first tested if the emotion‐focused condition elicited higher emotion exploration‐related outcomes and the solution‐focused condition higher collaboration‐related outcomes, and that there were no significant differences in the control variables perceived stress, difficulty, and problem significance between conditions. In addition, we expected and tested if genuine IS would be elicited in both conditions, resulting in higher observed IS in comparison to pseudosynchrony.

Subsequently, the first aim of this study was to identify markers (i.e., modalities and facets) of IS in the two relational contexts. Our hypotheses were grounded in a theoretical account of IS as a mechanism for integrating the self with the other during social interaction (Gordon et al. 2024; Heggli et al. 2021; Hoehl et al. 2021; Hu et al. 2022; Mayo and Gordon 2020). Within this framework, IS is thought to function by blurring the boundaries between self and other, thereby reducing interpersonal variability. For example, greater integration may be beneficial in contexts requiring high alignment (e.g., empathically listening to someone's emotional experience), whereas greater segregation may be advantageous in contexts requiring flexibility (e.g., generating novel ideas during problem‐solving). Based on this perspective, we hypothesized:

Hypothesis 1 *The emotion‐focused context would involve a greater need for dyadic integration, reflected in higher strength of physiological IS*.

This expectation aligns with prior work linking physiological IS to empathic processes (Marci et al. 2007). In contrast, we hypothesized:

Hypothesis 2 *The solution‐focused context would involve greater dyadic segregation, reflected in higher IS flexibility across modalities*.

Consistent with theoretical accounts suggesting that flexibility supports the incorporation of novel, self‐generated contributions in collaborative interactions (Gordon et al. 2024; Wood et al. 2021). Furthermore, we hypothesized:

Hypothesis 3 *The emotion‐focused condition would be characterized by more client‐leading dynamics*.

Hypothesis 4 *The solution‐focused condition would show more therapist‐leading dynamics*.

This prediction was informed by psychotherapy theory. Client‐centered and emotion‐focused psychotherapy approaches emphasize therapist nondirectiveness and position the client as the primary driver of the therapeutic interaction (Rogers 1946). In contrast, cognitive‐behavioral approaches are more structured and place the therapist in a more directive role within the interaction (Kazantzis and Dobson 2022). Additionally, as brainstorming may require more rapid shifts in coordination, we expected:

Hypothesis 5 *Greater switching between leading and following dynamics in the solution‐focused condition*.

The second aim of this study was to explore the relation between different IS markers and the two components of the WA across relational contexts. Building on prior work showing that physiological IS is more strongly associated with the emotional bond component of the WA in imagery‐based treatment contexts (Bar‐Kalifa et al. 2019), and evidence linking physiological IS to empathy (Marci et al. 2007), we hypothesized:

Hypothesis 6 *Greater physiological IS strength would be more strongly associated with the emotional bond component of the WA in the emotion‐focused condition*.

Conversely, we hypothesized:

Hypothesis 7 *Higher flexibility across modalities would be more strongly associated with the collaborative component and goal‐oriented component of the WA in the solution‐focused condition*.

It is consistent with the idea that flexible coordination supports collaborative, task‐focused engagement. All hypotheses were preregistered (https://doi.org/10.17605/OSF.IO/CE48Y).

In addition to these theoretical aims, this study directly addresses current methodological challenges in the quantification of IS by comparing multiple modalities and facets, and calculating different quantification metrics, within a unified experimental framework. This allows for a systematic evaluation of how different IS indices converge or diverge across relational contexts, thereby informing the selection of appropriate methods for specific experimental designs and data characteristics of IS. Accordingly, we conclude with methodological recommendations regarding the selection of IS modalities and facets, as well as the experimental manipulation of relational context.

## 2 Method

### 2.1 Participants

Participants (*N* = 168) were recruited from the psychology and pedagogical sciences bachelor's (44% first year, 16% second year, 18% third year) and master's programs (22%) of the Radboud University in the Netherlands. Participants were paired in dyads based on three criteria: No existing bond (i.e., being unacquainted), same gender, and same language preference (English or Dutch). Choosing same‐gender dyads is common practice to reduce gender‐related variability (see for example, Ramseyer and Tschacher 2011; Tschacher et al. 2014), as previous research has shown that IS can differ as a function of dyad gender composition (Li et al. 2021). There were 16 male dyads, 67 female dyads, and 1 nonbinary dyad in the final sample (Age: *M* = 22, SD = 4.5). Out of these, 36% had the conversations in Dutch. Participants were compensated with either a 15 euros voucher or 1.5 course credits. The recruitment and data collection took place at the Behavioral Sciences Institute of the Radboud University. The study was approved by the ethics committee of the Radboud Faculty of Social Sciences (ECSW‐2023‐088). This study was preregistered on the Open Science Framework (https://osf.io/ce48y/overview), and deviations are reported in the Supporting Information (Table S1).

### 2.2 Questionnaires

#### 2.2.1 The Working Alliance Inventory (WAI)

The WAI (Hatcher and Gillaspy 2006) for client and therapist were used both as a manipulation check (for the context manipulation) and as the primary outcome measure of perceived relationship quality. The short (12‐item version) was adapted following recommendations by Falkenström et al. (2015) to tailor it for repeated measures and specific interactions: We changed the verb tense of items from present to past (e.g., “I believe my therapist liked me”), and the Likert‐scale ranged from 0 “not at all” to 5 “*completely*”. The average scores of the goal and task subscales (8 items) and the average score of the bond subscale (4 items) were used as collaboration and emotional bond measures, respectively. The collaboration component reflects the perceived agreement on goals and tasks, whereas the emotional bond reflects the perceived trust, acceptance, and mutual confidence between the client and therapist. Internal consistencies in the present sample were good: WAI collaboration (therapist *α* = 0.88; client *α* = 0.89), WAI bond (therapist *α* = 0.76; client *α* = 0.87).

#### 2.2.2 The Barrett–Lennard Relationship Inventory (BL)

The Empathic Understanding subscale was used as context manipulation check in two versions: *Myself‐toward‐the‐Other* for the therapist and *Other‐toward‐Self* for the client (Barrett‐Lennard 1978). Both subscales measure perceived therapist empathy. Each version consists of 12 items and was scored on a 5‐point Likert scale (0 “not at all” to 5 “*completely*”). In line with the WAI above, the verb tense of items was changed from present to past (e.g., “My therapist usually sensed or realized what I was feeling”). Internal consistencies in the present sample were good (therapist *α* = 0.75; client *α* = 0.88).

#### 2.2.3 The Facilitative Interpersonal Skills (FIS)

The FIS‐Empathy item and the FIS‐Collaboration item were also used to assess the context manipulation (Santos et al. 2022). These two items measure client's perceptions of the therapist's ability to empathize and collaborate, respectively. Each item is rated on a 5‐point Likert scale, ranging from poor skill demonstration (e.g., empathy: “My therapist offered no sense that he/she actually understood my experience, or even distorted my experience”) to ideal skill demonstration (e.g., empathy: “My therapist offered appropriate reflections demonstrating that he/she was not only listening but also had an exceptional understanding of what I was/am experiencing”).

#### 2.2.4 The Helping Skills Measure (HSM)

The HSM‐Exploration subscale was used to assess emotion‐focus, and the HSM‐Action subscale to assess solution‐focus (Hill and Kellems 2002). The Exploration subscale contains four items that measure whether the therapist helped the client explore their feelings and thoughts. We adapted this subscale to focus on feelings only, by removing references to ‘thinking’ from the questions: “My therapist asked questions to help me explore what I was feeling”. Items were rated on a 6‐point Likert scale (1 “*strongly disagree*” to 6 “*strongly agree*”) with the instruction to use the option “*strongly disagree*” only for therapist behavior that did not occur in the past interaction. The Action subscale contains five items (all reverse coded) that measure collaboration on changing a problem (e.g., “My therapist did not help me think about changes I could make in my life”). The average score of each subscale was calculated. Internal consistencies were good (HSM‐Exploration *α* = 0.83; HSM‐Action *α* = 0.87).

#### 2.2.5 Perceived Difficulty, Stress, and Significance of the Problem

Perceived difficulty and stress were measured as a manipulation check for both participants using two self‐constructed items (“How stressed were you?” and “How difficult was this conversation?”). In addition, the client was asked how significant the problem they shared was (“How important to you was the problem you shared?”). All three items were rated on a 10‐point Likert scale (0 “not at all” to 10 “*extremely*”).

### 2.3 Client–Therapist Role‐Play Interaction Task

Participants were instructed to imagine themselves in a real therapy room during a first therapy session. From this point onward, we use the terms “client” and “therapist” to refer to the roles adopted by participants in the role‐play. Therapists were instructed to start the conversation by inviting the client to share the problem they wanted to discuss. Clients shared a different problem in each conversation. Both client and therapist received written instructions tailored per interaction to manipulate the conversational goal: Exploring emotions (*emotion‐focused interaction*) vs. brainstorming solutions (*solution‐focused interaction*):

#### 2.3.1 Emotion‐Focused Interaction

Therapists were informed that the goal of the conversation was to help their clients *explore their feelings* and to empathize with them. We also instructed them not to attempt to fix their clients' problems. Therapists were asked to help their clients express their feelings regarding their problems, explore an emotionally charged moment they identified together, and try to validate their clients' feelings. Therapists also watched a 3‐min animated video explaining empathy. To ensure their engagement, clients also received instructions stating that the goal of the conversation was to explore their feelings, and they were given three emotion‐focused questions to self‐reflect on their problem as preparation.

#### 2.3.2 Solution‐Focused Interaction

Therapists were informed that the goal of the conversation was to help their clients *brainstorm about concrete means* to solve their problem. They were asked to help their clients reflect on how they handled their problem, identify a goal to achieve together, and brainstorm solutions to achieve that goal. Therapists also watched a 3‐min animated video explaining how to collaborate with a client to agree on goals and solutions together. Clients also received instructions stating that the goal of the interaction was to brainstorm about solutions to their problem with the therapist and they were given three solution‐focused questions to self‐reflect as preparation. Role‐play instructions and videos are available at the OSF repository (https://osf.io/5r9gv/overview).

### 2.4 Procedure

Participants filled in their preferred language and availabilities while signing up to participate in the study. They were then contacted by phone and/or email to pair them into dyads and schedule a lab visit. They were asked to fill in several trait questionnaires (e.g., attachment style) before their visit, which are beyond the scope of the current paper. To prepare for their lab visit, they were asked to think of two real problems they would like to share in case they were assigned the client role. On the day of the experiment, participants were randomly assigned to play either the client or therapist role by drawing one of two concealed pieces of paper labeled “client” or “therapist”. Each participant remained in the assigned role throughout both interaction conditions. The study followed a within‐subject design with all dyads having two consecutive 13‐min interactions: one emotion‐focused and one solution‐focused. The order of the interactions was counterbalanced by alternating the order between successive dyads. The client's problems were randomly assigned to the two interactions. Participants were asked to sign a nondisclosure agreement stating they would not share the problems discussed with anyone to protect their privacy.

Participants were seated at a 45‐degree angle to one another for the video recording, and the physiological sensors were attached to them. They were both given the written role‐play instructions along with a laptop and a headset for the therapist to watch the video. After they finished, they were informed they would have 13 min for their first conversation and that they would hear a knock on the door when they had 1 min left as a sign to wrap up. The researcher monitored the conversation (without sound) from an adjacent experimenter room via the video recording. Participants were informed that the researcher could see them through the camera but could not listen to what they were saying to make sure they felt safe.

After the first interaction was over, participants were asked to complete all the self‐report questionnaires in Qualtrics using a laptop. After they finished, they were handed the role‐play instructions to prepare for the second interaction. Then, participants were reminded about the knock before beginning the second interaction. Afterwards, participants completed all the self‐report questionnaires again. A sliding room divider was always placed between participants when they were filling in questionnaires and while attaching the physiological sensors for privacy. The total duration of the study was 90 min.

### 2.5 Interpersonal Synchrony

#### 2.5.1 Data Recording

The interactions were video recorded using a Logitech HD Pro Webcam recording via OBS Studio (OBS Project, n.d.) with a video‐codec of H264‐ MPEG‐4 AVC, 1920 × 1080, 30 fps. Electrocardiogram (ECG) and electrodermal activity (EDA) were recorded using Biopac MP‐160 with a 1000 Hz sampling rate. The ECG was acquired with a high pass filter of 0.05 Hz and a gain of 2000. Disposable ECG electrodes were attached according to an adapted Lead II configuration by placing the active leads on the lower left rib and right collarbone, and the reference lead on the left collarbone (Quigley et al. 2024). EDA was acquired with a low‐pass filter set at 10 Hz, and a gain of 5 μS/V. Two Ag‐AgCl electrodes with isotonic gel were placed on the medial phalanx of the index and middle finger of the nondominant hand and cables were taped to the participant's arm to minimize motion artifacts.

#### 2.5.2 Data Preprocessing

##### 2.5.2.1 Motion Data

Movement was quantified using motion energy analysis (MEA; Ramseyer 2020b). MEA works by calculating the changes in pixel values over time by comparing consecutive video frames for a Region of Interest (ROI). Assuming the only movement within the ROI is from the participant, the MEA outputs a coarse‐grained measure of moment‐to‐moment general movement magnitude. For each participant, the ROI covered the upper body from the chair's seating area to the arms, excluding the head, given that upper‐body IS has been found to predict session‐level WA ratings (Ramseyer and Tschacher 2014). The 45‐degree seating arrangement allowed unobstructed visibility of both participants' upper bodies while capturing both participants in the same frame. This seating arrangement is common in the majority of IS research (e.g., Ramseyer and Tschacher 2011, 2014). The raw motion energy time series for clients and therapists were extracted and preprocessed using the *rMEA* package (Kleinbub and Ramseyer 2021). This entailed smoothing, using a moving average window of 0.5 s, rescaling using the Standard Deviation (SD), standardizing using *z*‐scores, and removing extreme outliers exceeding 10 SD in the data. The resulting *z*‐score standardized time series with 1 s resolution were used in subsequent analysis.

##### 2.5.2.2 High Frequency Heart Rate Variability

ECG preprocessing was conducted in Python using Jupyter Notebook (Project Jupyter, n.d.) and the NeuroKit2 library (Makowski et al. 2021). The ECG signal was first cleaned using NeuroKit2's automated filtering procedures. R‐peaks were then detected using a fully automated algorithm (Emrich et al. 2023), with fallback to an alternative method (Nabian et al. 2018) when detection failed. Heart period (HP; interbeat interval) time series were derived from successive R–R intervals. Following general recommendations for R‐R interval editing (Peltola 2012), artifact detection and correction were performed using a hybrid automated procedure combining median absolute deviation (MAD)‐based detection, percentage change thresholds (25%–35%), physiological plausibility bounds (0.35–1.6 s), and pattern‐based detection of abrupt swings and nonphysiological ramps. Additionally, R‐R intervals were visually inspected, and artifact selection was manually adjusted when artifacts were not adequately identified by the automated procedure. Isolated outliers were corrected with linear interpolation, and continuous outlier segments were corrected using local interpolation based on surrounding valid intervals. The number and percentage of corrected intervals were quantified for each recording. To ensure data quality, extended interpolated segments (> 3 s) were considered unreliable, and recordings with more than 5% of R‐R intervals corrected were excluded (*n* = 1). The corrected R‐R series was interpolated using cubic spline interpolation to obtain a uniformly sampled time series at 4 Hz (Quigley et al. 2024). HF‐HRV was calculated using a short‐time Fourier transform approach based on procedures described in Gates et al. (2015). Spectral estimates were computed using a 32 s sliding window with 1 s steps (128 samples per window). Within each window, power spectral density was estimated using Welch's method, combined with multitaper spectral estimation to stabilize spectral estimates. Power spectra across tapers were averaged to obtain a single spectrum per window. HF‐HRV was quantified as spectral power within the 0.12–0.40 Hz frequency band, reflecting vagally mediated heart rate variability associated with respiratory sinus arrhythmia (Pham et al. 2021). HF power values were log‐transformed prior to analysis, resulting in a time series of log HF‐HRV values sampled at 1 s resolution across each interaction.

##### 2.5.2.3 Electrodermal Activity

EDA signals were also processed in Python using NeuroKit2. Raw signals were minimally filtered with a 1 s median filter, a 1 s moving average, and a 10 Hz fourth‐order Butterworth low‐pass filter to reduce noise. Artifacts were automatically identified using rule‐based thresholds (implausible amplitudes: outside 0.01–100 μS, abrupt step changes: > 0.3 μS within 30 ms, and excessive slopes: > 5 μS/s). Short artifact segments (≤ 3 s) were linearly interpolated, whereas longer segments were marked unreliable during manual screening. Three dyads were dropped in both conditions due to poor signal quality (> 5% of unreliable segments) in either the client or therapist recording, and one additional dyad was dropped only from the solution‐focused condition. Client and therapist signals were resampled to a shared 4 Hz time grid and segmented into time‐locked 5 s windows (1 s step size). Within each window, we extracted both Average and Slope EDA values. EDA_Average_ reflects the overall tonic level of sympathetic arousal, whereas EDA_Slope_ captures dynamic changes in the signal over time and may reflect spontaneous fluctuations in the tonic level or nonspecific skin conductance responses (Boucsein et al. 2012). Although prior work has primarily focused on EDA_Slope_ (Marci et al. 2007; Palmieri et al. 2018), this study examined both signals to explore potential differences in their sensitivity to contextual changes. Finally, both EDA_Average_ and EDA_Slope_ time series were *z*‐standardized resulting in two EDA time series at 1 s resolution.

#### 2.5.3 Interpersonal Synchrony Computation

##### 2.5.3.1 Surrogate Datasets

Time‐locked cleaned therapist and client time series (movement, HF‐HRV, EDA_Slope_, and EDA_Average_, described above) were used for all IS computations. To assess whether observed IS exceeded chance levels, surrogate datasets were generated to serve as a control comparison (Moulder et al. 2018). The *surrogate* function from the *nonlinearTseries* package in R was used to apply a fast Fourier transform. This method constructs artificial time series that preserve each signal's power spectrum (i.e., its autocorrelation structure) while randomizing phase information (i.e., meaningful alignment between signals). This allows testing whether observed IS arises from autocorrelations (noise) or genuine interaction. For each dyad and condition, 100 surrogate time series were generated separately for client and therapist data. These surrogate datasets were then used to estimate pseudosynchrony by applying the same IS analysis procedure as used for the observed data (see below).

##### 2.5.3.2 Parameter Sweeping and Pseudosynchrony

Windowed Cross‐Lagged Correlation (WCLC) was used to compute IS, where cross‐correlations are computed within overlapping sliding windows. A typical WCLC matrix is a time‐by‐lag matrix in which columns represent time lags and rows represent successive overlapping windows across the interaction. The number of rows/windows depends on the interaction duration, window size, and step size. Parameter choice impacts the sensitivity of IS detection, especially the window size as it determines the number of observations per segment (Behrens et al. 2020). Given inconsistencies in the literature (Atzil‐slonim et al. 2023), we adopted a combined theory‐informed and data‐driven approach (as preregistered) by first comparing the three most commonly used window sizes in the literature and then choosing the one that maximized the difference between observed and surrogate IS, irrespective of condition. For each modality, three window sizes were tested (movement: 10, 30, 60 s; HF‐HRV: 16, 32, 60 s; EDA: 15, 30, 60 s), with corresponding step sizes proportional (~1/3) to the window length (movement: 4, 10, 20 s; HF‐HRV: 5, 10, 20 s; EDA: 5, 10, 20 s). Within each window, cross‐correlations were computed across modality‐specific maximum lags (movement: 5; HF‐HRV: 7; EDA: 10 s), reflecting the different temporal dynamics of each signal. For example, HF‐HRV primarily reflects respiratory sinus arrhythmia, with a typical adult respiration cycle ranging from approximately 4–7 s (Fisher et al. 2016), while EDA responses typically reach their peak approximately 3–5 s after activation, with recovery periods lasting several additional seconds (Posada‐Quintero and Chon 2020).

For each dyad, WCLC matrices were computed separately for observed and surrogate time series using identical procedures. For parameter sweeping, we compared average observed IS with average surrogate IS (both computed using three window sizes per modality). For each dyad, IS was calculated by averaging correlation coefficients across all time windows separately for each lag. WCLC was computed for both observed and surrogate time series using the same method. For the surrogate data, IS values from 100 surrogate pairs were averaged to produce a single surrogate IS profile per dyad, window size, and lag. Next, two‐sided paired‐sample *t*‐tests were conducted comparing observed and surrogate IS for each window size using average absolute IS across lags. See Figure 1 for an example from the HF‐HRV analysis, with window sizes on the *y*‐axis, time lags on the *x*‐axis, and cells representing *t*‐statistics comparing observed and surrogate IS. Results from these *t*‐tests for all modalities indicated that all tested window sizes showed significantly higher observed IS compared to surrogate data (see Supporting Information for detailed results). To maintain consistency across modalities, we selected the 60s window size for all following analyses. Each modality was assigned its modality‐specific maximum lag; therefore, all references to time lags below correspond to the maximum viable lag within that modality.

**FIGURE 1:**
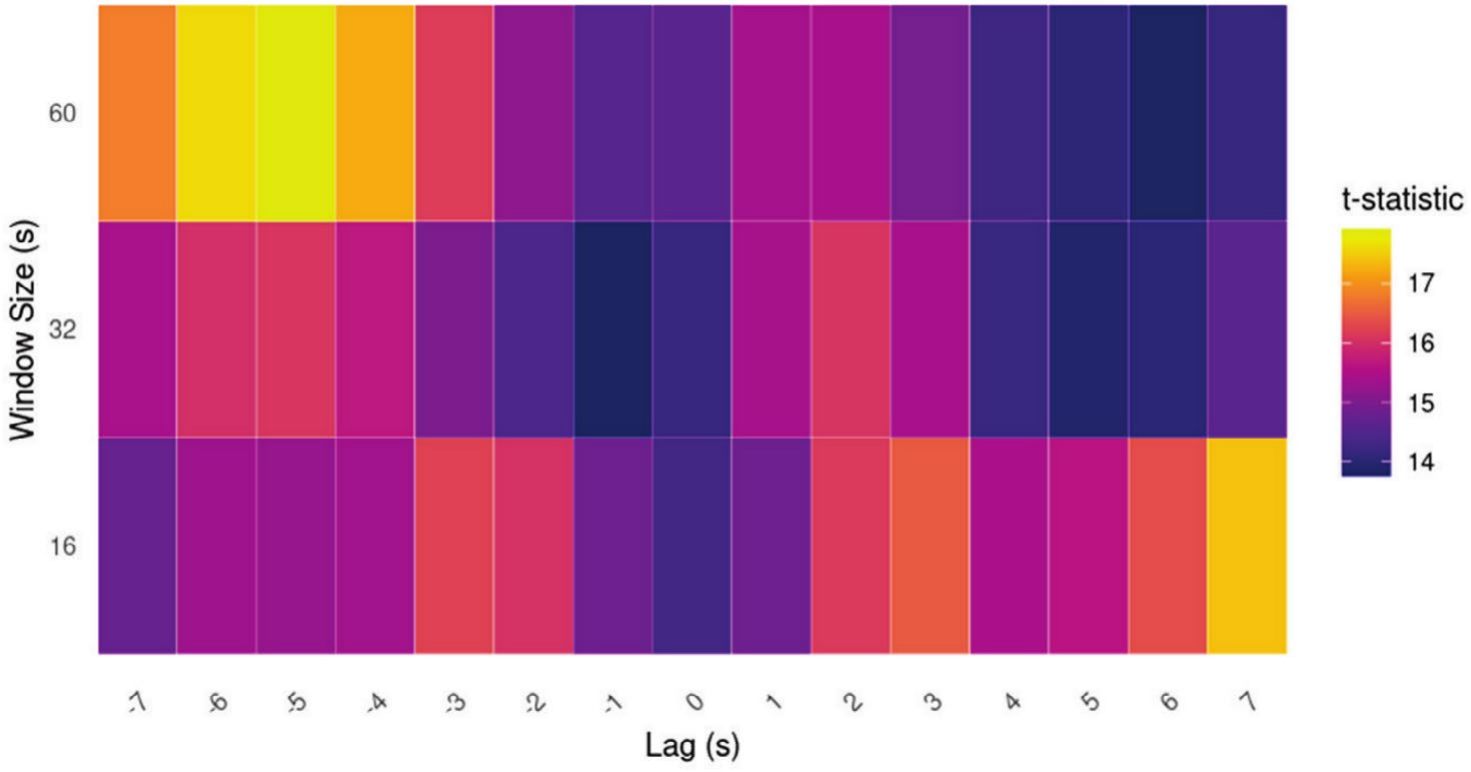
Paired *t*‐tests results from the parameter sweeping for HF‐HRV.

##### 2.5.3.3 Peak Picking and Synchrony Facets

Using the selected parameters, WCLC matrices were computed per dyad and condition (for both observed and surrogate data) and Fisher *z*‐transformed. The WCLC matrices were used to extract the following six facets: (1) The mean of absolute Fisher *z*‐transformed cross‐correlations was used as a *global strength* measure reflecting the average correlation magnitude between client and therapist across all time lags (Ramseyer and Tschacher 2011). (2) The variance between all correlations was used as a *global flexibility* measure reflecting the total variability between client and therapist across all time lags (Gordon et al. 2024).

A peak‐picking function using a Loess‐smoothing algorithm was then employed on the raw WCLC matrices to identify the dominant lag within each time window, resulting in a vector with peak correlations and the time lags at which they occurred (Boker et al. 2002; Moulder et al. 2018). peak detection was applied to each windowed correlation after Loess‐smoothing across lags (span = 0.25) to reduce noise. The dominant peak was defined as the maximum absolute correlation, with a look‐ahead stopping parameter (Lsize = maximum lag) which determines how many successive search expansions of the lag window are allowed without finding a stronger peak before the search terminates. (3) The mean of absolute peak correlations was used as a *peak strength* measure reflecting the maximum strength of IS observed between client and therapist. (4) The SD of absolute peak correlations was used as a *peak flexibility* measure reflecting the amount of variation in peak correlations between client and therapist. (5) The average of the time lags of peak correlations was used to represent *dominance* in the *leading‐following dynamics* (positive = client leading, negative = therapist leading). (6) The SD of the time‐lags of peak correlations was used to represent *switching* in the *leading‐following dynamics* between the client and therapist (with higher values indicating higher switching).

### 2.6 Data Analysis

All statistical analyses were conducted in R (version 4.4.1) using linear mixed‐effects models (package lme4). See Supporting Information for packages version and reference lists. Dyad was treated as the primary sampling unit and included as a random intercept in all models. Condition was sum‐to‐zero coded and specified as a fixed factor (−1 = emotion‐focused, 1 = solution‐focused). When included, continuous predictors were centered. All models were estimated using restricted maximum likelihood (REML). To control for multiple testing, *p*‐values for condition effects were adjusted using the false discovery rate (FDR) procedure. Model assumptions were checked using the check_model function from the performance package. Overall, diagnostics indicated that model assumptions were adequately met. As preregistered, model comparisons between base and interaction models (explained below) were conducted using likelihood ratio tests and information criteria (AIC). Gender, age, and therapists' experience (i.e., years of study) were checked as control variables and none of them improved model fit. All codes for preprocessing and analyses will be available on OSF repository (https://osf.io/5r9gv/overview).

#### 2.6.1 Experimental Manipulation of Context

To test the experimental manipulation of relational context, a series of linear mixed‐effects models examined the effect of condition (emotion‐focused vs. solution‐focused) on client‐ and therapist‐rated variables (Base model: Outcome ~ Condition + (1 | Dyad)). FDR corrections were applied to the analyses within each hypothesis family (emotion exploration‐related outcomes, collaboration‐related outcomes, and control variables). Emotion exploration‐related outcomes included: WAI‐Bond, BL‐Empathy, Affective empathy, and Cognitive empathy, all rated by client and therapist, as well as client‐rated FIS‐Empathy and HSM‐Emotion exploration. Collaboration‐related outcomes included: WAI‐Collaboration, rated by client and therapist, as well as client‐rated FIS‐Collaboration and HSM‐action. Control variables included perceived stress and difficulty (rated by client and therapist), as well as client rated significance of the problem.

#### 2.6.2 Pseudosynchrony

To test whether observed synchrony exceeded chance levels, paired one‐sided *t*‐tests compared observed synchrony against surrogate synchrony for each modality across four synchrony facets (global strength, peak strength, global flexibility, and peak flexibility) within each condition. We did not compare facets indexing leading‐following dynamics to surrogate data, as these positional facets are interpreted only in the context of above‐chance global synchrony. FDR correction was applied separately for each modality and condition across the four synchrony facets (i.e., eight tests per modality).

#### 2.6.3 Contextual Differences in Interpersonal Synchrony

To test whether synchrony facets and modalities differed between conditions (Hypotheses 1, 2, 3, 4, 5), we conducted another set of linear mixed‐effects models with the different IS modalities and facets as outcome (Base model: IS ~ Condition + (1 | Dyad)). FDR correction was applied across facets per modality (i.e., correcting for six tests per modality). To check for potential order effects of the two conditions, we ran control analyses (interaction models) by adding sequence (A: emotion‐focused condition first; B: solution‐focused condition first), and the Condition × sequence interaction to the base models. The results reported below are from the base models (without sequence) unless stated otherwise, in cases where sequence affected the conclusions regarding condition effects.

#### 2.6.4 Relations Between Interpersonal Synchrony and Working Alliance

To investigate whether the relations between IS and the experienced relationship quality depended on the relational context (Hypotheses 6 and  7), we ran a series of mixed‐effect models to see whether the different IS modalities and facets predicted the experienced WAI (bond and collaboration, separately for clients and therapists) differently per condition (WA ~ Condition * IS + (1 | Dyad)). FDR correction was applied within theoretically defined facet families (strength, flexibility, leading‐following), separately for each person (client, therapist) and WA component (bond, collaboration). Each correction was applied to eight tests per family (4 modalities × 2 facets per family). We report below the models that showed significant results after FDR correction. The full list of results (2 roles × 2 subscales × 4 IS modalities × 6 IS facets = 96 models) is available as Supporting Information.

## 3 Results

### 3.1 Experimental Manipulation of Context

Descriptive results are reported in Table 1 and results from the linear mixed‐effects models are reported in Table 2. As expected, the emotion‐focused condition was associated with significantly higher therapist self‐rated empathy in comparison to the solution‐focused condition (BL, Affective Empathy, and Cognitive Empathy). Therapist empathy, as rated by the client (BL, FIS), was also high in the emotion‐focused condition, but not significantly higher than in the solution‐focused condition. Client‐rated emotional exploration of their problem (HSM‐Exploration) also did not differ significantly between conditions after correcting for multiple testing. Finally, experienced WAI‐Bond was high and did not differ significantly between conditions for both therapists and clients.

**TABLE 1:** Means and standard deviations for self‐report measures.

Role	Measure	Therapist		Client	
		Emotion‐focused *M* (SD)	Solution‐focused *M* (SD)	Emotion‐focused *M* (SD)	Solution‐focused *M* (SD)
Emotion exploration‐related	WAI‐Bond	4.48 (0.73)	4.51 (0.81)	4.44 (0.96)	4.45 (0.99)
	BL‐empathy	58.35 (6.50)	56.27 (6.39)	58.43 (9.36)	58.17 (8.54)
	Cognitive empathy	4.99 (0.96)	4.75 (0.85)	4.89 (1.03)	4.73 (1.02)
	Affective empathy	4.59 (1.13)	4.20 (1.10)	4.23 (1.24)	4.02 (1.32)
	HSM‐Exploration	—	—	4.26 (0.80)	4.02 (0.87)
	FIS‐empathy	—	—	4.04 (0.84)	3.95 (0.86)
Collaboration‐related	WAI‐collaboration	3.50 (0.99)	4.63 (0.78)	3.85 (1.02)	4.84 (0.80)
	HSM‐action	—	—	2.65 (0.99)	4.12 (0.66)
	FIS‐collaboration	—	—	3.82 (0.88)	4.12 (0.72)
Control	Stress	3.85 (2.21)	3.51 (2.16)	3.70 (1.94)	3.55 (2.13)
	Difficulty	3.99 (1.97)	3.64 (2.15)	4.08 (2.20)	3.75 (2.24)
	Significance	—	—	7.27 (1.92)	6.76 (1.91)

Abbreviations: BL, Barrett–Lennard Relationship Inventory; FIS, facilitative interpersonal skills; HSM, helping skills measure; *M*, mean; SD, standard deviation; WAI, Working Alliance Inventory.

**TABLE 2:** Results from the linear mixed‐effects models for the experimental manipulation check.

Measure family	Role	Measure	Estimate	SE	95% CI	*p*	*p* (FDR)	*R* ^2^ marginal	*R* ^2^ conditional	ICC
Emotion exploration‐related	Therapist	WAI bond	0.013	0.035	[−0.056, 0.083]	0.701	0.878	0.000	0.660	0.660
		BL empathy	−1.137	0.340	[−1.813, −0.461]	**0.** **001**	**0.** **010**	0.031	0.532	0.517
		Cognitive empathy	−0.131	0.056	[−0.242, −0.020]	**0.** **021**	**0.** **025**	0.021	0.364	0.350
		Affective empathy	−0.202	0.065	[−0.331, −0.074]	**0.** **002**	**0.** **010**	0.033	0.448	0.429
	Client	WAI‐Bond	0.006	0.039	[0.071, 0.083]	0.878	0.878	0.000	0.737	0.737
		BL‐empathy	−0.131	0.459	[−1.043, 0.781]	0.776	0.878	0.000	0.560	0.560
		Cognitive empathy	−0.083	0.056	[−0.195, 0.028]	0.141	0.141	0.007	0.502	0.498
		Affective empathy	−0.101	0.068	[−0.237, 0.034]	0.141	0.283	0.006	0.524	0.521
		HSM‐exploration	−0.121	0.054	[−0.227, −0.014]	**0.** **027**	0.072	0.021	0.322	0.308
		FIS‐empathy	−0.042	0.054	[−0.148, 0.065]	0.440	0.704	0.002	0.335	0.334
Collaboration‐related	Therapist	WAI‐collaboration	0.567	0.060	[0.448, 0.686]	**< 0.001**	**< 0.001**	0.286	0.465	0.251
	Client	WAI‐collaboration	0.494	0.057	[0.380, 0.608]	**< 0.001**	**< 0.001**	0.226	0.494	0.346
		HSM‐action	0.737	0.058	[0.621, 0.853]	**< 0.001**	**< 0.001**	0.436	0.547	0.197
		FIS‐collaboration	0.149	0.052	[0.045, 0.252]	**0.** **005**	**0.** **008**	0.033	0.318	0.295
Control	Therapist	Difficulty	−0.185	0.126	[−0.435, 0.066]	0.147	0.245	0.008	0.377	0.372
		Stress	−0.179	0.103	[−0.383, 0.026]	0.086	0.216	0.007	0.630	0.627
	Client	Difficulty	−0.167	0.136	[−0.438, 0.105]	0.225	0.281	0.006	0.368	0.365
		Stress	−0.077	0.100	[−0.277, 0.122]	0.443	0.443	0.001	0.591	0.591
		Significance	−0.256	0.125	[−0.505, −0.007]	**0.** **044**	0.216	0.018	0.292	0.279

*Note:* Positive estimates reflect higher scores in the solution‐focused condition relative to the emotion‐focused condition; negative estimates reflect the opposite. Bold formatting indicates statistical significance at *p < 0.05.

Abbreviations: BL, Barrett–Lennard Relationship Inventory; CI, confidence intervals; FDR, false discover rate; FIS, facilitative interpersonal skills; HSM, helping skills measure; ICC, intraclass correlation coefficient; SE, standard error; WAI, Working Alliance Inventory.

Conversely, in line with expectations, the solution‐focused condition was associated with significantly higher therapist and client‐rated WAI‐Collaboration, and clients also rated therapists' collaborative skills and actions (FIS‐Collaboration and HSM‐Action) higher in this condition. Finally, as expected, there were no significant differences between conditions in perceived stress or difficulty, either for therapists or for clients, nor for client‐rated problem significance.

Together these results show that our experimental manipulation was effective at a subjective level, although the condition differences were more notable in the collaboration‐related measures than the emotion exploration‐related measures, and the experienced difference in empathy was stronger for therapists than clients.

### 3.2 Interpersonal Synchrony

In the subsections below, we report results organized by modality. First, we report the comparison between observed IS and pseudosynchrony for each modality (movement, HF‐HRV, and EDA) and four synchrony facets (global strength, peak strength, global flexibility, and local flexibility). Table 3 shows the test statistics, and the full descriptives are reported in the Supporting Information (Table S2). Next, we report the condition differences for each modality and the six facets (the four above plus leading‐following dynamics: dominance and switching). Fixed effects results from the base models are presented in Table 4. In general, the fixed effects (condition) explained a small proportion of total variance (marginal *R* ^2^), whereas the full models including random effects (for dyads) showed higher explanatory power (conditional *R* ^2^). Results from the interaction models (including sequence) are reported in the text when relevant.

**TABLE 3:** Results from *t*‐test for observed vs. pseudosynchrony.

Modality	Condition	Emotion‐focused			95% CI	Solution‐focused			95% CI
	Facet	*t* (df)	*p* (FDR)	*d*		*t* (df)	*p* (FDR)	*d*	
Movement	Global strength	10.81 (83)	**< 0.001**	1.18	[0.90, 1.46]	10.01 (83)	**< 0.001**	1.09	[0.82, 1.36]
	Peak strength	10.99 (83)	**< 0.001**	1.20	[0.92, 1.48]	10.00 (83)	**< 0.001**	1.09	[0.82, 1.36]
	Global flexibility	2.80 (83)	**0.** **005**	0.31	[0.09, 0.52]	2.82 (83)	**0.** **006**	0.31	[0.09, 0.53]
	Peak flexibility	3.10 (83)	**0.** **003**	0.34	[0.12, 0.56]	2.43 (83)	**0.** **013**	0.27	[0.05, 0.48]
H‐HRV	Global strength	3.06 (82)	**0.** **009**	0.34	[0.11, 0.56]	3.66 (82)	**< 0.001**	0.40	[0.18, 0.62]
	Peak strength	2.51 (82)	**0.** **014**	0.28	[0.06, 0.49]	3.28 (82)	**0.** **002**	0.36	[0.14, 0.58]
	Global flexibility	2.51 (82)	**0.** **014**	0.28	[0.06, 0.49]	4.58 (82)	**< 0.001**	0.50	[0.27, 0.73]
	Peak flexibility	1.27 (82)	0.124	0.14	[−0.08, 0.36]	3.16 (82)	**0.** **002**	0.35	[0.12, 0.57]
EDA (Slope)	Global strength	7.92 (80)	**< 0.001**	0.88	[0.62, 1.14]	8.86 (79)	**< 0.001**	0.99	[0.72, 1.26]
	Peak strength	6.58 (80)	**< 0.001**	0.73	[0.48, 0.97]	7.61 (79)	**< 0.001**	0.85	[0.59, 1.11]
	Global flexibility	8.88 (80)	**< 0.001**	0.99	[0.72, 1.25]	7.48 (79)	**< 0.001**	0.84	[0.58, 1.09]
	Peak flexibility	8.32 (80)	**< 0.001**	0.92	[0.66, 1.18]	7.41 (79)	**< 0.001**	0.83	[0.57, 1.08]
EDA (Average)	Global strength	9.13 (80)	**< 0.001**	1.01	[0.74, 1.28]	9.11 (79)	**< 0.001**	1.02	[0.75, 1.29]
	Peak strength	9.15 (80)	**< 0.001**	1.02	[0.75, 1.28]	9.08 (79)	**< 0.001**	1.01	[0.74, 1.28]
	Global flexibility	10.23 (80)	**< 0.001**	1.14	[0.85, 1.41]	9.26 (79)	**< 0.001**	1.03	[0.76, 1.30]
	Peak flexibility	7.48 (80)	**< 0.001**	0.83	[0.58, 1.08]	6.94 (79)	**< 0.001**	0.78	[0.52, 1.02]

*Note:* df = *n*—1. Bold formatting indicates statistical significance at *p < 0.05.

Abbreviations: CI, confidence intervals; *d*, Cohen's *d* effect size; FDR, false discovery rate; *M*, mean; SD, standard deviation.

**TABLE 4:** Results from linear mixed‐effects models for condition differences in IS.

Modality	Facet	Condition estimate	SE	95% CI	*t*	*p*	*p* (FDR)	*R* ^2^ marginal	*R* ^2^ conditional	ICC
Movement (df = 168)	Global strength	0.004	0.002	[0.000,0.009]	2.019	**0.** **045**	0.142	0.008	0.676	0.674
	Peak strength	0.004	0.002	[0.000,0.008]	1.998	**0.** **047**	0.142	0.007	0.689	0.687
	Global flexibility	0.000	0.001	[−0.001,0.001]	−0.684	0.495	0.721	0.002	0.151	0.149
	Peak flexibility	−0.001	0.002	[−0.004, 0.003]	−0.357	0.722	0.721	0.001	0.229	0.229
	Dominance	0.028	0.077	[−0.124, 0.180]	0.359	0.720	0.721	0.001	0.205	0.204
	Switching	0.031	0.020	[−0.008, 0.070]	1.549	0.123	0.246	0.012	0.148	0.137
HRV (df = 162)	Global strength	0.000	0.003	[−0.005, 0.006]	0.077	0.938	0.938	0.000	0.009	0.009
	Peak strength	0.000	0.003	[−0.006, 0.007]	0.140	0.889	0.938	0.000	0.033	0.033
	Global flexibility	0.001	0.001	[−0.001, 0.002]	0.624	0.534	0.938	0.002	NA	NA
	Peak flexibility	0.002	0.002	[−0.001, 0.005]	1.275	0.204	0.938	0.010	NA	NA
	Dominance	0.049	0.075	[−0.100, 0.198]	0.652	0.515	0.938	0.002	0.082	0.080
	Switching	0.016	0.003	[−0.053, 0.086]	0.467	0.641	0.938	0.001	0.185	0.184
EDA Slope (df = 157)	Global strength	0.002	0.002	[−0.001, 0.006]	1.224	0.223	0.334	0.008	0.127	0.120
	Peak strength	0.003	0.003	[−0.003, 0.010]	0.985	0.326	0.391	0.005	0.123	0.119
	Global flexibility	0.001	0.001	[0.000, 0.002]	1.743	0.083	0.249	0.013	0.333	0.324
	Peak flexibility	0.001	0.002	[−0.003, 0.004]	0.395	0.693	0.693	0.001	0.463	0.462
	Dominance	−0.200	0.103	[−0.403, 0.003]	−1.950	0.053	0.249	0.021	0.106	0.087
	Switching	−0.065	0.046	[−0.157, 0.027]	−1.397	0.164	0.328	0.009	0.255	0.248
EDA Average (df = 157)	Global strength	−0.001	0.003	[−0.008, 0.005]	−0.372	0.710	0.867	0.000	0.525	0.525
	Peak strength	−0.001	0.003	[−0.007, 0.006]	−0.167	0.867	0.867	0.000	0.342	0.342
	Global flexibility	0.000	0.001	[−0.003, 0.002]	−0.198	0.843	0.867	0.000	0.487	0.487
	Peak flexibility	0.000	0.002	[−0.003, 0.004]	0.297	0.767	0.867	0.000	0.273	0.273
	Dominance	0.032	0.089	[−0.144, 0.209]	0.364	0.717	0.867	0.001	0.050	0.050
	Switching	−0.034	0.045	[−0.123, 0.054]	−0.768	0.444	0.867	0.003	0.179	0.176

*Note:* df = *n*‐1; Positive estimates reflect higher scores in the solution‐focused condition relative to the emotion‐focused condition; negative estimates reflect the opposite. Bold formatting indicates statistical significance at *p < 0.05.

Abbreviations: CI, confidence intervals; FDR, false discovery rate; NA, not applicable (model produced a singular fit warning); SE, standard error.

#### 3.2.1 Movement Interpersonal Synchrony

##### 3.2.1.1 Observed vs. Pseudosynchrony

Results showed above‐chance levels of observed movement IS for all facets in both conditions, but with a clear distinction between strength‐ and flexibility‐based facets, with larger effect sizes for strength (*d*s = 1.09–1.19) than for flexibility (*d*s = 0.26–0.33), see Table 3.

##### 3.2.1.2 Condition Differences in Interpersonal Synchrony

For movement IS, neither *strength* nor *flexibility* facets (global or peak) showed significant condition differences after FDR correction in the base models (see Table 4). However, accounting for sequence revealed a significant condition × sequence interaction for global strength, *b* = −0.007, SE = 0.002, *t*(162) = −3.19, 95% CI [−0.011, −0.002], *p* = 0.002. Model comparison indicated that this interaction model fit significantly better than the base model, *χ* ^2^(2) = 10.63, *p* = 0.005, and showed lower ΔAIC = 6.63. While the main effect of condition was also significant in this model, *b* = 0.004, SE = 0.002, *t*(162) = 2.15, CI [0.00, 0.01], *p* = 0.034, this effect was moderated by sequence (the presentation order of conditions). The main effect of sequence was not significant, *b* = 0.005, SE = 0.005, *t*(162) = 1.01, CI [−0.01, 0.02], *p* = 0.317. Post hoc pairwise comparisons of estimated marginal means (EMMs) showed that dyads who started with the solution‐focused condition (sequence B), showed significantly lower movement global strength in the subsequent emotion‐focused condition (*M* = 0.14, CI [0.12, 0.15]) compared to the solution‐focused condition (EMM = 0.16, CI [0.14, 0.17]), *p* < 0.001. In contrast, dyads who started with the emotion‐focused condition (sequence A), did not differ in movement IS global strength between conditions (emotion‐focused EMM = 0.16, CI [0.15, 0.18]; solution‐focused EMM = 0.16, CI [0.14, 0.17]), *p* = 0.469. See Figure 2.

**FIGURE 2:**
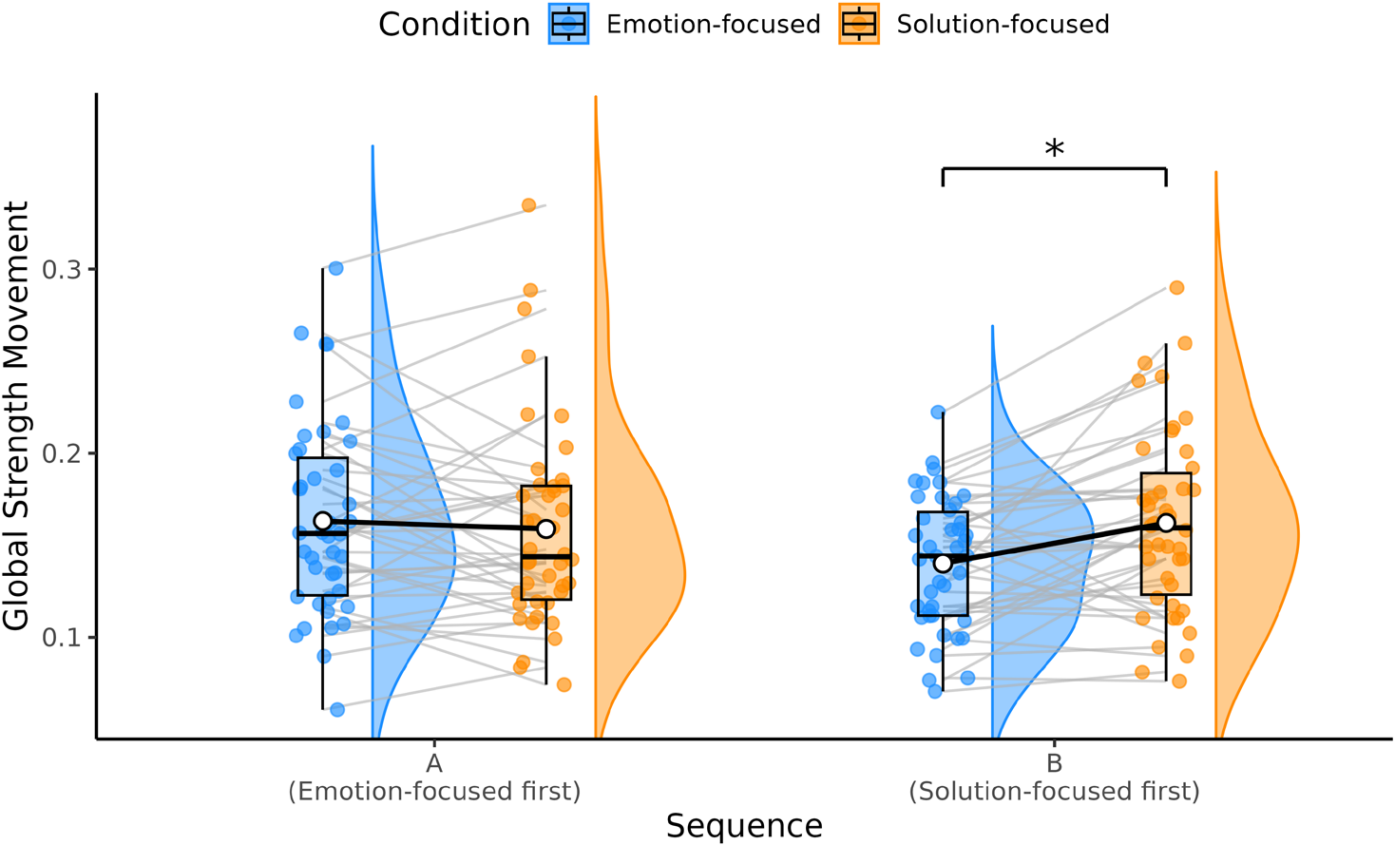
Movement (global strength) by condition and sequence. Colored points represent dyads, with gray lines connecting repeated observations. Half‐violins depict the distribution of values, and boxplots summarize the median and interquartile range. White points and black lines indicate condition means. *p < 0.001.

In addition to reduced mean synchrony, between‐dyad variability in the emotion‐focused condition was lower when it followed the solution‐focused condition. The estimated standard deviation for the emotion‐focused condition decreased from 0.037 (sequence A; administered first) to 0.008 (sequence B; administered second), suggesting that dyads responded more homogeneously to the emotion‐focused condition when it was preceded by the solution‐focused condition.

The same moderation pattern emerged for peak strength (See Supporting Information for Figures). The Condition × sequence interaction was significant, *b* = −0.006, SE = 0.0019, *t*(162) = −3.16, CI [−0.010, −0.002], *p* = 0.002. As with global strength, dyads who began with the solution‐focused condition showed reduced peak strength movement IS in the subsequent emotion‐focused condition (emotion‐focused EMM = 0.15, CI [0.14, 0.16]; solution‐focused: EMM = 0.17, CI [0.16, 0.18], *p* < 0.001), whereas no condition differences emerged for dyads who started with the emotion‐focused condition (emotion‐focused EMM = 0.17, CI [0.16, 0.19]; solution‐focused EMM = 0.17, CI [0.15, 0.18]), *p* = 0.471. Results for *leading‐following dynamics* showed that both dominance (mean peak lag) and switching (SD peak lag) did not differ significantly between conditions for movement IS (see Table 4) and including sequence in the model did not change these results.

#### 3.2.2 EDA Interpersonal Synchrony

##### 3.2.2.1 Observed vs. Pseudosynchrony

Results showed above‐chance levels of EDA IS across all facets in both conditions, with slightly larger effect sizes for EDA_Average_ (strength: *d*s = 1.01–1.02; flexibility facets: *d*s = 0.78–1.14) than EDA_Slope_ (strength facets: *d*s = 0.73–0.99; flexibility facets: *d*s = 0.84–0.99), see Table 3.

##### 3.2.2.2 Condition Differences in Interpersonal Synchrony

None of the *strength* or *flexibility* facets differed significantly between conditions for either EDA_Slope_ or EDA_Average_, see Table 4.

For *leading‐following* dynamics of both EDA_Slope_ and EDA_Average_, dominance and switching did not differ between conditions after FDR correction in the base models (see Table 4). However, for EDA_Slope_ dominance, accounting for sequence in the model provided a significantly better fit to the data, *χ* ^2^(2) = 7.68, *p* = 0.021, ΔAIC = 3.68. Although the within‐dyad effect of condition (emotion‐focused vs. solution‐focused) was not statistically significant, *b* = −0.20, SE = 0.10, *t*(155) = −1.96, CI [−0.40, 0.00], *p* = 0.051, the between‐dyad main effect of sequence was significant, *b* = 0.28, SE = 0.11, *t*(155) = 2.56, CI [0.07, 0.49], *p* = 0.011. Dyads who completed the emotion‐focused conversation first (sequence A) showed positive lag values (*M* = 0.28, SE = 0.15), suggesting client leading dynamics overall, whereas dyads who completed the solution‐focused conversation first (sequence B) exhibited negative lag values (*M* = −0.28, SE = 0.15), or therapist leading dynamics overall. See Figure 3. The interaction was not significant, *b* = −0.11, SE = 0.10, *t*(155) = −1.09, CI [−0.31, 0.09], *p* = 0.274.

**FIGURE 3:**
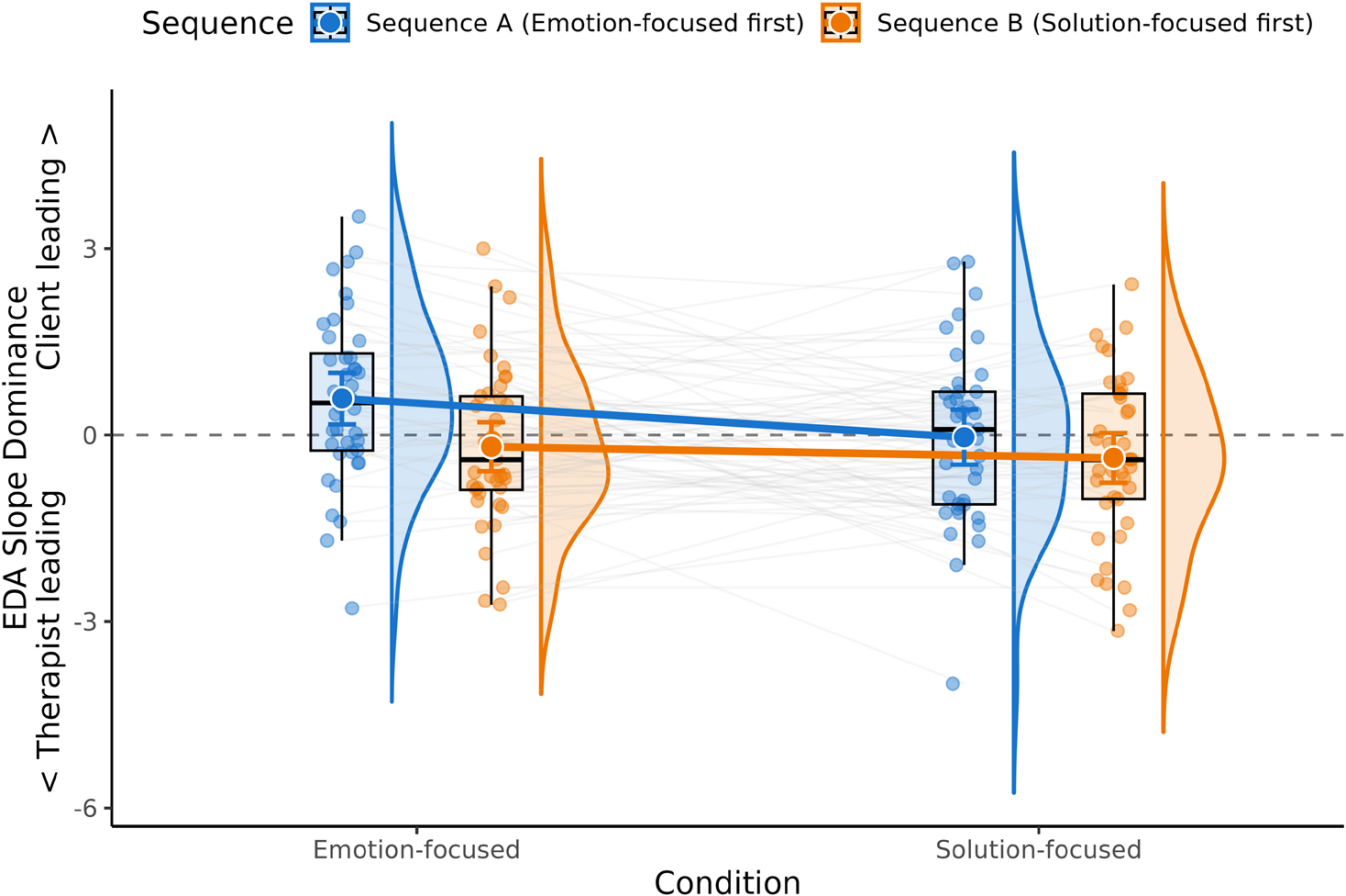
EDA_Slope_ dominance main effect of sequence. Individual datapoints represent dyads, with gray lines connecting repeated observations across conditions (nonsignificant within‐dyads condition effect). Colored lines indicate sequence means with 95% confidence intervals (significant between‐dyad effect). Half‐violins depict the distribution of values, and boxplots summarize the median and interquartile range.

#### 3.2.3 HF‐HRV Interpersonal Synchrony

##### 3.2.3.1 Observed vs. Pseudosynchrony

Results showed above‐chance levels of HF‐HRV synchrony with similar effect sizes for strength facets in both conditions (*d*s = 0.27–0.40), but slightly larger effects for flexibility facets in the solution‐focused (global: *d* = 0.50; peak: *d* = 0.34) than the emotion‐focused condition; (global: *d* = 0.27; peak: *d* = *n*.s.), see Table 3.

#### 3.2.4 Condition Differences in Interpersonal Synchrony

None of the *strength*, *flexibility*, or *leading‐following dynamics* facets differed significantly between conditions for HF‐HRV IS (see Table 4), and accounting for sequence did not change these results. For global flexibility and peak flexibility, a singular fit occurred because the dyad random intercept variance was estimated at zero (ICC ≈0), indicating negligible between‐dyad variability. Models without the random intercept yielded identical fixed effects; therefore, fixed‐effects‐only results are reported (ICC and conditional *R* ^2^ not applicable; Figner et al. 2024).

In summary, pseudosynchrony results across modalities and facets showed above‐chance levels of IS in both conditions. Effect sizes were similar for global and peak strength facets but differed between strength and flexibility facets. Condition differences were observed only for movement strength facets and EDA_Slope_ dominance and were moderated by sequence.

### 3.3 Relations Between Interpersonal Synchrony and Experienced Working Alliance

To investigate whether the relations between IS and the experienced relationship quality depended on the relational context, we ran another series of mixed‐effect models to see whether the different IS modalities and facets predicted the experienced WAI differently per condition. For both client‐ and therapist‐rated WAI collaboration, we found no significant main effects of IS, nor any significant interactions between IS and condition across modalities and facets (See Table 5 for full results of models with significant effects, and Supporting Information for all other models). In other words, we found no relations between IS and the collaborative component of the WAI.

**TABLE 5:** Fixed effects for models with significant condition × synchrony interactions.

Model	Parameter	Estimate	SE	CI low	CI high	*t*	*p*	*p* (FDR)
Client bond EDA_Slope_ dominance	(Intercept)	4.473	0.100	4.274	4.673	44.569	< 0.001	—
	Condition	0.006	0.041	−0.075	0.087	0.142	0.887	—
	EDA_Slope_ dominance	−0.028	0.040	−0.107	0.051	−0.707	0.481	0.769
	Condition: EDA_Slope_ dominance	0.104	0.037	0.030	0.178	2.777	**0.** **007** **	**0.** **053**
Therapist bond EDA_Slope_ dominance	(Intercept)	4.494	0.078	4.339	4.648	57.890	< 0.001	—
	Condition	0.001	0.036	−0.069	0.072	0.041	0.967	—
	EDA_Slope_ dominance	−0.025	0.034	−0.093	0.043	−0.737	0.462	0.912
	Condition: EDA_Slope_ dominance	0.092	0.032	0.028	0.156	2.840	**0.** **005** **	**0.** **043** *
Therapist bond Movement global strength	(Intercept)	4.490	0.076	4.338	4.642	58.847	< 00.001	—
	Condition	0.011	0.034	−0.058	0.079	0.306	0.760	—
	Movement global strength	0.649	1.211	−1.742	3.040	0.536	0.593	0.929
	Condition: Movement global strength	1.915	0.745	0.434	3.395	2.570	**0.** **012** *	**0.** **047** *
Therapist bond Movement peak strength	(Intercept)	4.490	0.076	4.338	4.642	58.834	< 00.001	—
	Condition	0.010	0.034	−0.058	0.078	0.295	0.769	—
	Movement peak strength	0.835	1.328	−1.787	3.457	0.629	0.530	0.929
	Condition: Movement peak strength	2.089	0.807	0.485	3.693	2.588	**0.** **011** *	**0.** **047** *

*Note:* Bold formatting indicates statistical significance at *p < 0.05.

Abbreviations: CI, confidence intervals; FDR, false discovery rate; SE, standard error.

* *p* < 0.05.

** *p* < 0.01.

For the therapist‐rated WAI bond, results showed a significant Condition × Synchrony interaction for both movement strength facets (global: *B* = 1.92, peak: *B* = 2.09), see Table 5. Both strength facets showed similar results as illustrated in Figure 4. Follow‐up simple slopes analyses showed that movement global strength was relatively positively associated with the experienced bond in the solution‐focused condition, *b* = 2.56, SE = 1.33, *t*(154.75) = 1.92, *p* = 0.056, 95% CI [−0.07, 5.20], and negatively in the emotion‐focused condition, *b* = −1.27, SE = 1.53, *t*(154.75) = −0.83, *p* = 0.410, CI [−4.29, 1.76], although neither of the slopes within the conditions was significant. For movement peak strength, the slope was significantly positively related to the experienced bond in the solution‐focused condition, *b* = 2.92, SE = 1.46, *t*(156.13) = 2.00, *p* = 0.048, CI [0.03, 5.82], and relatively negative but not significant in the emotion‐focused condition, *b* = −1.25, SE = 1.67, *t*(156.13) = −0.75, *p* = 0.453, CI [−4.55, 2.04]. These results show that higher movement IS strength was associated with a higher therapist‐rated bond in the solution‐focused condition than the emotion‐focused condition.

**FIGURE 4:**
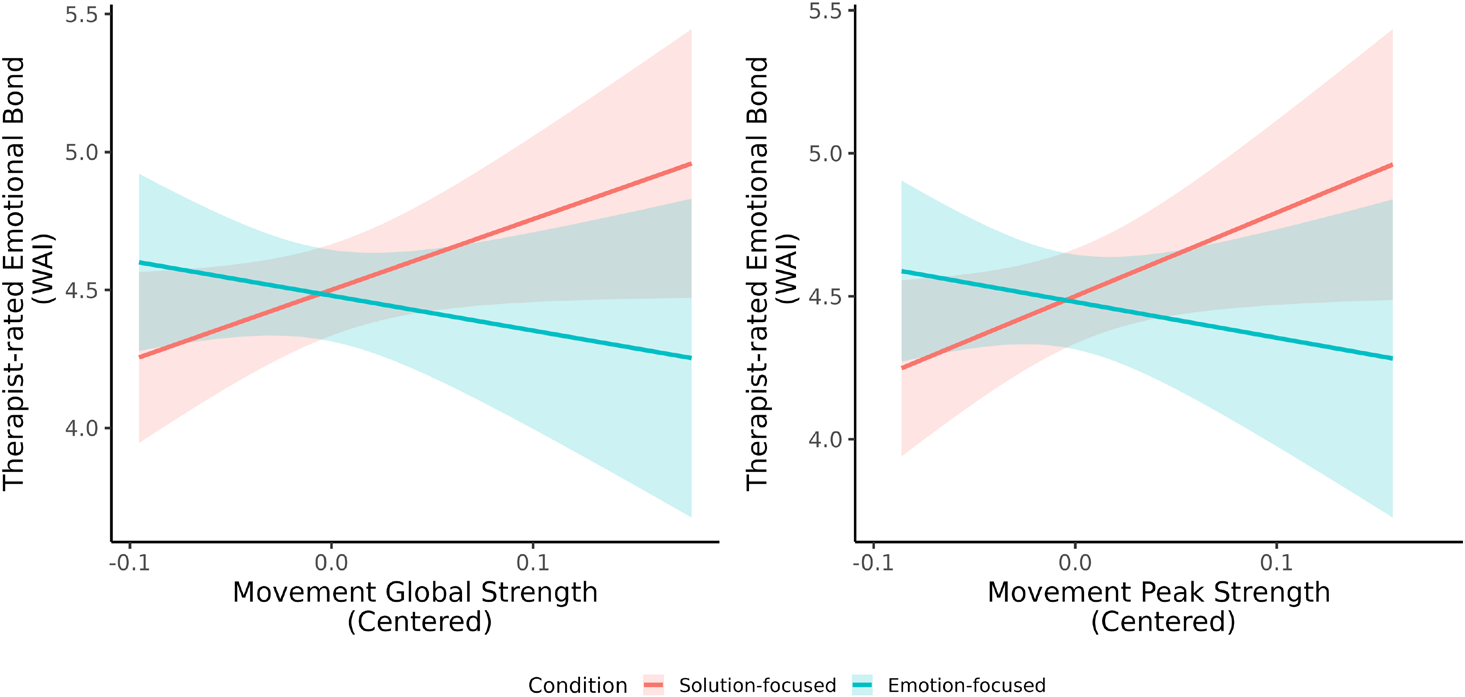
Interaction between movement IS (global and peak strength) and condition for therapist‐rated WAI emotional bond.

Additionally, for both client‐ and therapist‐rated WAI‐Bond, we found a significant condition × IS interaction for EDA_Slope_ dominance (Therapist: *B* = 0.09; Client: *B* = 0.10), see Table 5. Because negative and positive values of EDA_Slope_ dominance reflect qualitatively different patterns (negative = therapist‐leading; positive = client‐leading), we examined condition differences at low (−1 SD), mean, and high (+1 SD) levels of EDA_Slope_ dominance. Figure 5 illustrates the similar observed patterns for client and therapist ratings. For the client‐rated WAI‐Bond, there was a significant condition difference at low dominance (therapist‐leading), *b* = −0.27, SE = 0.13, *t*(89.72) = −2.08, *p* = 0.040, with higher bond scores in the emotion‐focused than in the solution‐focused condition. No difference was found at mean dominance, *b* = 0.01, SE = 0.08, *t*(78.30) = 0.14, *p* = 0.887. At high dominance (client‐leading), the pattern was reversed, *b* = 0.30, SE = 0.13, *t*(89.40) = 2.26, *p* = 0.026, with higher bond scores in the solution‐focused condition. The same pattern was observed for therapist‐rated WAI‐Bond: low IS, *b* = −0.25, SE = 0.11, *t*(92.88) = −2.18, *p* = 0.032; mean IS, *b* = 0.01, SE = 0.07, *t*(78.56) = 0.04, *p* = 0.887. *p* = 0.967; high IS, *b* = 0.26, SE = 0.11, *t*(92.51) = 2.23, *p* = 0.028. Overall, these results suggest that for EDA_Slope_ dominance, more therapist‐leading was associated with an increased perceived bond (for both therapists and clients) in the emotion‐focused condition, whereas client‐leading was associated with an increased perceived bond in the solution‐focused condition.

**FIGURE 5:**
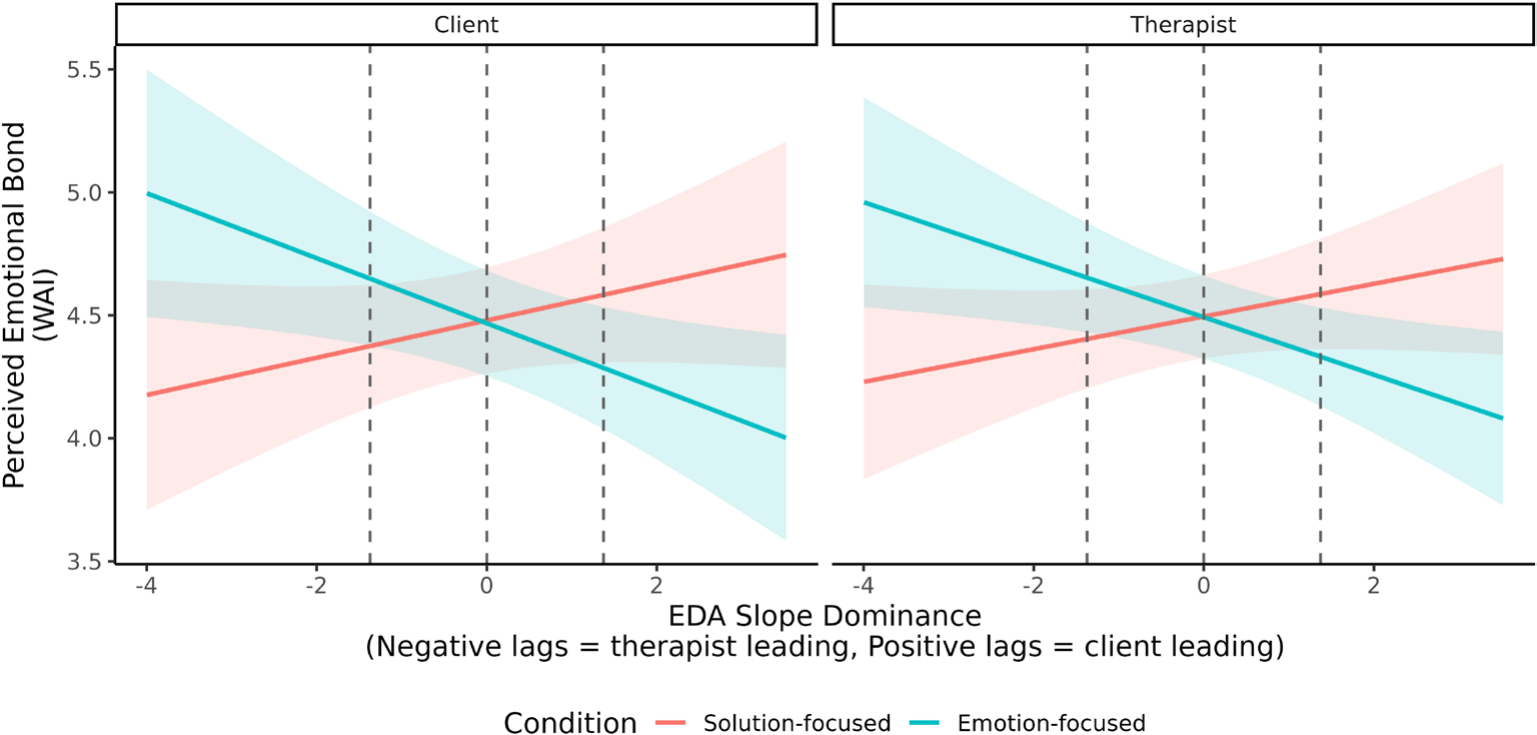
EDA_Slope_ dominance × condition interaction for client‐ and therapist‐rated emotional bond. Dashed vertical lines indicate the focal values at which condition differences were examined in the follow‐up analysis (−1 SD, mean, and +1 SD of EDA_Slope_ dominance).

## 4 Discussion

This study examined whether different modalities and facets of IS changed across emotion exploration‐focused vs. solution‐focused relational contexts, and if they were related to different components of the client and therapist perceived working alliance (WA) movement, high‐frequency heart rate variability (HF‐HRV), and electrodermal activity (EDA) IS modalities were quantified using WCLC to extract facets of IS reflecting strength (peak and global), flexibility (peak and global), and leading–following dynamics (dominance and switching). We experimentally manipulated relational context by assigning different conversation goals (i.e., emotional exploration vs. brainstorming solutions) in our client–therapist role play task. The subjective ratings supported the effectiveness of our manipulation: As expected, we observed higher ratings on emotion exploration‐related measures in the emotion‐focused condition, and higher ratings on collaboration‐related measures in the solution‐focused condition. The effects on emotion exploration‐related measures were larger for therapists than clients. There were no differences between conditions in perceived stress, difficulty, or significance of the problem discussed. For IS, we found higher levels of observed IS compared to pseudosynchrony across all modalities and facets in both conditions. This is an important check, showing evidence of above‐chance level (i.e., genuine) IS, even though we used client–therapist role‐play tasks with relatively short conversations. These findings align with studies from other domains that found evidence of IS in experimental setups (Tschacher et al. 2014). In the sections below, we first address the results related to the two theoretical aims of this study, followed by methodological recommendations for future experimental research on IS.

### 4.1 Contextual Differences in Interpersonal Synchrony, and Effects of Dyadic History

The first aim of our study was to identify markers of IS across different relational contexts, that is, how context (the two conditions in our experimental set‐up) affects different modalities and facets of IS. Contrary to our expectations (Hypotheses 1, 2, 3, 4, 5), we did not observe an effect of condition on IS for any of the modalities and facets in our base models after correcting for multiple testing. This is inconsistent with previous literature reporting differences in IS depending on relational context (e.g., competitive vs. cooperative contexts) in laboratory studies (Chanel et al. 2012) or emotion‐focused vs. cognitive interventions in psychotherapy research (Bar‐Kalifa et al. 2019). It is also not aligned with recent theoretical frameworks emphasizing the importance of context in shaping IS dynamics (Gordon et al. 2024; Wood et al. 2021). Several methodological factors may explain the absence of expected effects (e.g., parameter optimization choices, multiple testing, or the effectiveness of the manipulation), which we discuss in the methodological recommendations section below. Here, we consider a complementary theoretical explanation concerning the conceptualization of relational context.

In our study design, we counterbalanced the order of conditions, which conversation goal participants started with, between‐dyads to experimentally control for sequence effects. However, when accounting for sequence in our analyses, we observed significant interaction effects between sequence and condition on some IS patterns. This suggests that the effects of relational context (condition) cannot be fully isolated from past interactions through counterbalancing. This makes sense when sequence is interpreted as *dyadic history*, in line with complex systems perspectives in which prior states (here: the first conversation) function as contextual constraints that shape future interaction dynamics (Juarrero 2023). This also aligns with frameworks describing coordination dynamics as metastable (Kelso 2012, 2022). Within this perspective, coordination is both flexible and history‐dependent, such that prior interactional states constrain the range of patterns that can emerge. Accordingly, the initial interaction may have acted as a constraint, shaping the dyad's coordination dynamics in the subsequent interaction, and influencing the effect of relational context.

Consistent with this interpretation, the first notable effect of dyadic history (i.e., sequence) was an interaction with condition for movement IS, specifically in global and peak strength. Dyads that performed the solution‐focused interaction first, showed lower (and less variable, see Figure 2) IS in the subsequent emotion‐focused condition, whereas dyads that started with the emotion‐focused condition did not show significant condition differences. A tentative explanation for this unexpected finding is that after collaborating to find solutions, interaction partners may have developed a clearer sense of how to coordinate with one another, which could make subsequent interactions more stable and predictable, and high levels of IS less necessary. This interpretation aligns with research suggesting that one potential function of IS is to facilitate prediction through alignment, making others' behavior easier to anticipate and reducing variability within social interactions (daSilva and Wood 2024; Diana and Kret 2025; Hoehl et al. 2021). From this perspective, self‐other integration, or the temporary blurring of boundaries, may be driven by the need to improve prediction of the interaction partner, with social bonding emerging as a secondary consequence rather than a primary driver (Diana and Kret 2025).

Second, we observed a main effect of dyadic history (i.e., sequence) for EDA_Slope_ dominance. Dyads who began with the emotion‐focused condition showed greater client‐leading dynamics overall, whereas dyads who began with the solution‐focused condition showed greater therapist‐leading dynamics overall, that is, in the absence of a condition effect. These findings suggest that conversation goals may shape coordination patterns through dyadic history (between‐dyads), even when changes in relational context do not show immediate condition effect (within‐dyads). Identifying the level at which relational contexts exert their influence is important (i.e., whether influence is more pronounced within‐dyads or between‐dyads), as research increasingly seeks to identify the contextual factors that shape IS (Gordon and Bartsch 2026; Zilcha‐Mano 2025). Additionally, it is important to note that this dyadic effect on dominance was specific to EDA_Slope_ and did not generalize across modalities. This suggests that variation in leading‐following dynamics may be more pronounced in SNS IS than in movement IS or PNS IS.

### 4.2 Relationship Between Interpersonal Synchrony and Experienced Working Alliance

The second aim of this study was to explore the relation between different IS markers and the two components of the WA (i.e., emotional bond vs. collaboration) across two conditions. We did not find support for our hypotheses that strength of physiological IS would relate more strongly to the emotional bond in emotion‐focused contexts (Hypothesis 6), and that IS flexibility (across modalities) would relate more strongly to collaborative set in solution‐focused contexts (Hypothesis 7).

We did however find that higher movement IS strength was associated with relatively higher therapist ratings of the emotional bond in the solution‐focused condition compared to the emotion‐focused condition. This differs from prior findings showing that physiological IS (EDA strength) was positively linked to the emotional bond in more emotion‐focused (imaginary) therapy segments, but not in more solution‐focused (CBT‐like) segments (Bar‐Kalifa et al. 2019). Both studies demonstrate a link between IS and the emotional bond but differ in the modality and context in which this association emerges. Interestingly, EDA_Slope_ dominance was also associated with the emotional bond (see below), but not with the collaborative component of the WA. Together, these results support the idea that IS may be associated with emotional bond across multiple modalities, in line with perspectives that conceptualize social bonding as one key function of IS (daSilva and Wood 2024), but also tentatively suggest that the patterns are more modality‐and context‐specific than previously assumed. As these specific patterns remain difficult to interpret and may reflect study‐specific factors, replication is needed.

As mentioned earlier, we also observed an interaction between condition and EDA_Slope_ dominance for emotional bond ratings. Specifically, in dyads that exhibited more *client‐led* EDA_Slope_ dynamics, both therapists and clients reported a stronger emotional bond in the *solution‐focused* condition, whereas dyads characterized by more *therapist‐led* EDA_Slope_ dynamics reported a stronger emotional bond in the *emotion‐focused* condition. One way to interpret this reversed pattern is in terms of task demands versus adaptive coordination within the dyad (Nowak et al. 2017). For example, although emotion‐focused therapy may naturally require clients to lead more—a task demand—(Rogers 1946), emotional bond was rated higher in dyads where therapists led more on EDA_Slope_. One possible explanation is that higher relationship quality is related to therapist responsiveness, i.e., the ability of the therapist to flexibly adjust behavior to meet the client's needs in each moment (Caspar 2024; Caspar et al. 2005). Such responsiveness can entail a deviation from primary task demands in favor of adaptive coordination to dynamics of the dyad.

However, in the absence of a clear theoretical account explaining these patterns, this interpretation remains speculative. Future research is needed to replicate these findings and to more systematically examine how different modalities of IS relate to distinct components of the WA across contexts.

### 4.3 Methodological Recommendations

Beyond the two main aims outlined above, this study also addressed current methodological challenges in the quantification of IS by comparing multiple IS modalities and facets within a unified experimental framework. In the section below, we provide methodological recommendations based on observations of how IS in different modalities and facets converged or diverged across relational contexts in our study. The section is subdivided into recommendations regarding IS modalities and facets, and the experimental manipulation of relational context. All recommendations are summarized in Box 1.

#### 4.3.1 Modalities

We observed several differences across IS modalities, with a particularly notable distinction within the EDA modality itself. Specifically, we observed a condition effect for EDA_Slope_ but not for EDA_Average_. This finding highlights how different quantifications of the same physiological modality can capture distinct aspects of IS. In this study, the cleaned EDA signal was analyzed using 5 s windows with a 1 s moving step. EDA_Average_ was computed as the mean value within each window, reflecting tonic skin conductance level (SCL), whereas EDA_Slope_ was calculated as the linear slope within each window, capturing the change rate in EDA over time. Although both measures reflect SNS activity (Posada‐Quintero and Chon 2020), EDA_Average_ may capture more stable, tonic levels of SNS activation, while EDA_Slope_ may capture more dynamic fluctuations (e.g., non‐specific skin conductance responses). Consistent with this notion, only EDA_Slope_ showed differences between conditions in this study. In addition, we also observed differences in the sources of explained variance. Specifically, EDA_Average_ showed near‐zero marginal *R* ^2^ (i.e., variance explained by fixed effects, i.e., condition) alongside higher conditional *R* ^2^ (variance including random effects, i.e., dyad), suggesting that it captured little variance related to our contextual manipulation and instead primarily reflected stable differences between dyads. Based on these findings, we suggest that EDA_Slope_ may be better suited than EDA_Average_ for capturing state‐level variation in IS, such as contextual differences or leading–following dynamics. This recommendation is further supported by prior work (e.g., Marci and Orr 2006), which advocates the use of slope to reduce the influence of idiosyncratic drift (i.e., slow, person‐specific changes in baseline EDA).

BOX 1 **Methodological recommendations for future research.**

**Table:** 

Modalities − Use EDA_Slope_ when the goal is to capture state‐level variation and use EDA_Average_ for trait‐level − Account for the influence of speech on HF‐HRV − Report window size selection procedure and optimization criteria
Facets − Use global strength and flexibility facets when not interested in leading following dynamics − Use peak strength and flexibility when interested in leading following dynamics
Relational context − Manipulate several contextual factors instead of isolating one − Design paradigm with several (> 2) condition phases and distinguish micro‐level from interaction‐level goals − Account for dyadic history (e.g., sequence effects)

Similar to EDA_slope_, HF‐HRV was extracted using a preprocessing approach that emphasized shared short‐term variation by differencing each time point from the previous one (Gates et al. 2015). Despite this approach, we observed relatively little variability in HF‐HRV IS, suggesting there was little variation to be captured. This was reflected in the mixed‐effects models, which produced singular fit warnings, indicating that the variance of the random intercept approached zero, meaning that the dyads did not differ much in HF‐HRV flexibility IS. Moreover, the small effect sizes for flexibility‐based facets (in comparison to pseudosynchrony), suggest that the observed IS minimally exceeded noise levels. One explanation for this could be that HF‐HRV is highly sensitive to breathing dynamics, and that conversational speech substantially alters respiratory rhythms (Quigley et al. 2024). As a result, part of the HF‐HRV signal may reflect physiological influences of speech (e.g., breathing patterns during turn‐taking), introducing noise in the signal within participants and masking IS dynamics.

Several approaches have been proposed to control for breathing on HF‐HRV (Quigley et al. 2024). One option is to measure respiration and incorporate it into the analysis (Beda et al. 2007). However, this method is complex and may risk overcorrecting the signal, potentially removing meaningful variance (Tininenko et al. 2012). An alternative approach, that is more relevant to IS research, is to incorporate a matched talking baseline condition that approximates the respiratory demands of the experimental task (Tininenko et al. 2012). We therefore recommend that future research account for the influence of speech on HF‐HRV.

Another recommendation concerns parameter selection during the parameter sweeping procedure. In this study, we aimed to offer a reproducible and transparent approach for selecting and WCLC parameters (see Supporting Information for detailed results). Different parameters (especially window size) can substantially influence the sensitivity to detect IS, making it difficult to compare findings across studies and to synthesize results within the broader literature. In this study, we chose parameter sets based on their ability to maximize the detection of IS relative to surrogate data, independent of the experimental conditions. This approach was adopted to minimize the risk of *p*‐hacking, given that we aimed to test hypotheses about condition effects on IS. However, our approach may be more sensitive to *between*‐dyad differences in IS (Behrens et al. 2020), as it prioritizes parameters that maximize the overall detectability of IS across dyads. According to Behrens et al. (2020), it may be better to choose parameters that maximize differences between conditions within the same dyad, when the research goal is to detect *within*‐dyad changes in synchrony. We recommend that future studies clearly report their parameter selection procedure and where possible, compare the impact of different optimization approaches (e.g., prioritizing within‐ vs. between‐dyad variations).

#### 4.3.2 Facets

When examining the effect sizes of the comparisons between observed versus pseudosynchrony, *strength‐based facets* consistently showed the largest effects and global and peak measures were nearly identical to one another. In contrast, flexibility‐based facets showed substantially smaller effect sizes, somewhat greater differences between global and peak measures, and between conditions. Overall, these results suggest that the distinction between strength and flexibility may be more influential than the choice between peak vs. global measures, especially for strength. We further observed clear consistencies in the observed condition effects between global and peak measures. Based on these observations, we recommend *global* over peak‐based metrics, as peak‐based approaches require an additional peak‐detection step that introduces further parameter choices. Using global metrics, therefore, reduces analytical degrees of freedom and may enhance reproducibility. However, peak‐based metrics remain particularly useful when the goal is to extract leading‐following dynamics. peak‐based facets capture IS at the specific lag where coordination is strongest, allowing leading‐following dynamics to be quantified at the point of maximal coupling. In contrast, global facets summarize IS across the entire lag spectrum. Therefore, when interested in leading‐following dynamics, we recommend using peak‐based (rather than global) measures for strength and flexibility as well, as this ensures that all facets are based on the same moments of peak coordination, maintaining conceptual consistency across facets.

In this study, multiple IS facets were tested intentionally to allow systematic comparisons across measures. However, the required FDR correction for multiple testing has probably reduced statistical power. We therefore recommend that hypothesis‐driven studies aiming to identify IS markers select facets based on theoretical relevance, thereby limiting the need for extensive multiple comparison correction. On the other hand, more exploratory studies are still needed for comparing facets, to build a comprehensive understanding of different patterns of IS.

#### 4.3.3 Relational Context

Although the subjective ratings suggested that the relational context manipulation in both conditions was largely successful, the emotion‐focused condition resulted in higher ratings on emotion exploration‐related outcomes from the therapist's perspective more so than from the client's. One possible explanation for this divergence is the distinction between expressed empathy by the therapist and experienced empathy by the client. Therapists were explicitly instructed to express empathy, so their higher ratings likely reflect a genuine effort to do so. Clients may have recognized these attempts without actually feeling understood. This interpretation is supported by the finding that, prior to correcting for multiple testing, clients reported greater emotional exploration (HSM), while empathy itself was not significantly higher. Together, this suggests that the condition was experienced as more emotion‐focused, but not necessarily as more empathic. A potential way to strengthen the manipulation would be to align the client's problem with the conversational goal from the outset, for example, asking clients to bring an emotionally salient issue to the emotion‐focused condition and a more neutral, problem‐solving issue to the solution‐focused condition. Although this introduces the risk of biasing the subjective importance of the problems across conditions, it may produce a stronger and more ecologically valid relational context. This highlights a broader trade‐off between experimental control and the strength or authenticity of the manipulation in IS research attempting to capture natural social interactions in the laboratory.

We therefore *recommend* that laboratory research on dyadic IS move beyond attempts to isolate single contextual factors and instead examine how multiple contextual factors jointly contribute to the emergence of a particular interactional context. For example, manipulating not only the conversational goal (e.g., focusing on emotions), but also characteristics of the problem discussed (e.g., selecting emotionally salient problems), as both elements shape the task demands of the interaction. This perspective aligns with viewing context as a web of constraints (Juarrero 2023), in which individual factors cannot be fully isolated and experimental control may instead be achieved by strategically combining interacting contextual elements to shape the interaction. This is especially relevant as research on IS increasingly moves into laboratory settings (see for example, Guralnik et al. 2024; Tschacher et al. 2014; Zeng et al. 2025). Attempts to isolate individual components to maximize experimental control may then risk oversimplifying the processes that generate IS and limit the ability to observe the phenomenon as it naturally unfolds.

A further limitation concerns the external validity of our student sample. Participants were randomly assigned the roles of “client” and “therapist”. They differed in their level of psychology education, including how much “professional skills” training they had received. Although our control analyses indicated that this did not influence the present findings, the “therapists” did not receive formal psychotherapy training and therefore cannot be considered representative of practicing clinicians. Previous work has shown that trained psychotherapists exhibit higher observer‐rated empathy and greater electrodermal synchrony than psychologists without psychotherapy training and non‐therapists during simulated clinical interactions (Messina et al. 2013), suggesting that psychotherapy training may affect interpersonal coordination processes. Similarly, the “clients” did not receive formal psychiatric diagnoses and presented with nonclinical problems. Consequently, they cannot be considered representative of clinical populations. Previous studies have examined IS across different clinical populations, including individuals with depression (Goren et al. 2024; Jennissen et al. 2024), anxiety (Altmann et al. 2020), and autism (Cheng et al. 2017). However, little is still known about how different psychiatric diagnoses influence IS dynamics. Accordingly, the present findings should be interpreted as reflecting coordination dynamics that emerge during experimentally induced helping interactions rather than psychotherapy per se. While our experimental paradigm provided greater control over relational context and enabled causal inferences that are difficult to achieve in observational psychotherapy research, future studies should examine whether these findings generalize to trained therapists and real clients in naturalistic psychotherapy sessions. Other variables such as seating arrangement, session duration, clinical diagnosis, and treatment type could also then be taken into account. Additionally, in this study the entire interaction (13 min) was analyzed as a single unit, for both subjective ratings and IS, assuming a consistent higher order goal (e.g., emotion‐focused or solution‐focused). However, interactions likely contain multiple phases with different micro‐goals, such as listening to the client's problem before moving into emotional exploration or problem‐solving. It is possible that the early phases of the conversations (involving problem‐sharing) were similar across conditions, masking micro‐level condition differences in later stages as we measured averages across the whole conversation. We therefore *recommend* that future experimental research design interactions in ways that more clearly separate phases with distinct goals (e.g., listening vs. discussing). This would not only strengthen the manipulation but also increase the number of observations per dyad, enabling the modeling of temporal variation (random slopes) within dyads. In addition, future work may benefit from analytical approaches that capture how IS dynamically evolves over the course of an interaction (Likens and Wiltshire 2021), allowing researchers to link changes in coordination to meaningful interactional events or shifts in relational processes (Kleinbub et al. 2024; Van Eijndhoven et al. 2023).

Additionally, given the strong influence of dyadic history on relational context observed in our findings, we *recommend* that future research explicitly incorporate dyadic history into both the design and analysis of IS studies. One approach is to consider sequence as a theoretically meaningful factor, for example, by systematically varying interaction sequences to examine how different prior contexts shape subsequent IS. Another approach is examining IS and their functional effects across longer time scales in which real‐life relationships unfold (days, months, etc.). Such designs are increasingly feasible with advances in methods such as Ecological Momentary Assessment (EMA) and wearable technologies, which allow researchers to capture interactional dynamics as they unfold in naturalistic settings (Olthof et al. 2024).

## 5 Conclusion

Taken together, our findings demonstrate the feasibility of studying client–therapist interactions and IS in laboratory settings. However, effects of relational context only emerged after accounting for dyadic history (i.e., sequence), underscoring the need to carefully consider how relational context is conceptualized and operationalized. Specifically, differences in movement IS strength and EDA_slope_ dominance were moderated by dyadic history. In addition, these modalities were associated with perceived emotional bond and not with perceived collaboration, supporting the previously reported link between IS and relational bonding processes, although specific patterns differed between modalities and contexts. Beyond these findings, this study highlights several methodological considerations for advancing IS research. Measurement and preprocessing choices should align with the inference level of interest (e.g., within‐ or between‐dyad variation), and control for confounds such as speech effects on HF‐HRV. Transparent reporting of parameter selection, particularly window size optimization, is critical for reproducibility. Furthermore, the choice of IS *facet* should reflect the research question, with global measures suited for overall coordination and peak‐based measures for examining leading–following dynamics. Finally, relational context should be treated as multidimensional and temporally structured: Rather than isolating single factors, we recommend that future IS research manipulate multiple contextual elements, use multiphase designs, and explicitly account for dyadic history to better understand the functions of IS across contexts and time scales.

## Author Contributions

**Noha M. Yassen:** conceptualization, formal analysis, data curation, writing – original draft, investigation, project administration. **Jacobien M. van Peer:** supervision, writing – review and editing, conceptualization, project administration. **Ger P. J. Keijsers:** supervision, writing – review and editing, conceptualization, project administration. **Leentje Vervoort:** supervision. **Travis J. Wiltshire:** supervision, writing – review and editing, conceptualization.

## Conflicts of Interest

The authors declare no conflicts of interest.

## Supporting information


**Table S1:** Deviations from the preregistration.
**Figure S1:** Results of *t*‐tests comparing observed vs. surrogate movement IS.
**Figure S2:** Results of *t*‐tests comparing observed vs. surrogate EDA IS.
**Table S2:** Descriptive statistics for observed and surrogate IS across modalities, facets, and conditions.
**Figure S3:** movement (peak strength) by condition and sequence.
**Table S3:** Fixed effects for models predicting WAI from condition × IS: movement IS.
**Table S4:** Fixed effects for models predicting WAI from condition × IS: HF‐HRV IS.
**Table S5:** Fixed effects for models predicting WAI from condition × IS: EDA slope IS.
**Table S6:** Fixed effects for models predicting WAI from condition × IS: EDA average IS.
**Table S7:** Interpersonal synchrony predicting client‐rated empathy.

## Data Availability

The data that support the findings of this study are available on request from the corresponding author. The data are not publicly available due to privacy or ethical restrictions.
