# Supplementary material for: Interpersonal Synchrony During a Client–Therapist Role‐Play Interaction: The Role of Modalities, Facets, and Relational Context
Source: Psychophysiology. 2026 Nov 1;63(11):e70436. doi: 10.1111/psyp.70436 (PMC13724790; doi:10.1111/psyp.70436)
Supplement: Supplementary file 1 [file PSYP-63-e70436-s001.docx]

**Supplementary Materials**

**Table of contents**

1. Deviations from preregistration………………………………..………………………2
2. Parameter Sweeping results……………………………………………………………3
3. Descriptives Observed versus Pseudosychrony………………………………………..5
4. Condition Differences in Interpersonal Synchrony……………………………...…….6
5. Relations between Interpersonal Synchrony and Working alliance…………………...7
6. Relations between Interpersonal Synchrony and Client-rated therapist empathy…....13
7. Packages versions and references………………………………………….…………15

**1. Deviations from the preregistration**

This study was preregistered on the Open Science Framework (https://osf.io/ce48y/overview), and deviations alongside the reasons are reported below.

**Table S1**

|  | *Deviations From the Pre-registration* |
| --- | --- |
| **1** | **Data collection**  As data collection took significantly longer than planned, and intermediate data checks showed overall good data quality, data collection was stopped once the target sample (84) was reached, and dyads with incomplete or poor-quality data not replaced. This led to loss of 3 dyads in EDA data, and 1 from ECG data. |
| **2** | **Data collection**  We expanded the inclusion criteria from including only student from (Clinical psychology master students, or psychology bachelor students who have finished the second-year course Conversational Skills in Psychology) to including all students from the psychology and pedagogical sciences bachelor'. |
| **2** | **Data analysis**  Instead of within-subjects 2-s segment shuffling and using SUSY (Tschacher & Haken, 2019), we used a fast Fourier transform from the nonlinearTseries package in R to generate our surrogate data. Next, instead of correcting observed IS for Pseudosynchrony, we relied on the statistics of the tests comparing observed IS vs. Pseudosynchorny that IS was above chance level. This is a common approach in the broader synchrony literature (beyond psychotherapy research). |
| **3** | **Data preprocessing**  Instead of using RSAseconds (a MATLAB based discrete Fourier transform method; Gates et al., 2015)**,** we extracted HF-HRV using a short-time Fourier transform (STFT) in jupyter notebook, python, using scipy.signal.welch. The STFT method is more suitable for time-series analysis, and the Python allowed implementing the preprocessing of all signals within a single environment. |
| **4** | **Hypothesis testing**  **Aim 2: Relationship between IS and relationship quality**  Instead of focusing only on client-rated WAI (preregistered H2.1 and 2), we tested for both client and therapist rated WAI (as reported in the main text), as we deemed both perspectives relevant, although the client perspective is more predominant in psychotherapy research. In addition, we also tested relations between IS and empathy, but due to space limitations (and because WAI was our primary outcome measure) these results are only reported in the supplementary materials. |

**2. Parameter Sweeping results**

Parameter sweeping results for HF-HRV are shown in the manuscript (Figure 1). Below, results for movement IS and EDA IS are shown. For all modalities, all tested window sizes showed significantly higher observed IS vs. surrogate.

***2.1 Parameter Sweeping for movement Interpersonal Synchrony***

For parameter sweeping (as reported in the manuscript), we used the following parameters for movement IS: window sizes 10, 30, 60 seconds; corresponding step sizes 4, 10, 20 seconds, and maximum lag 5 seconds. Figure S1 shows the resulting t-statistics were visualized as a heatmap, where higher values indicate stronger deviations from chance synchrony. As shown in the figure, all parameter combinations yielded robust evidence for real synchrony exceeding surrogate synchrony, with consistently large t-values across lags. Larger window sizes (particularly 60 s) produced the strongest effects, suggesting more stable synchrony estimates at broader temporal scales, while shorter windows yielded slightly weaker but still substantial effects. Overall, these results indicate that the presence of interpersonal synchrony is highly robust to variation in WCLC parameter settings.

**Figure S1.**

*Results of t-tests Comparing Observed vs. Surrogate movement IS*

***2.2 Parameter sweeping for Electrodermal Activity Interpersonal Synchrony (same for EDA Slope and Average)***

For parameter sweeping (as reported in the manuscript), we used the following parameters for EDA IS: window sizes 15, 30, 60 seconds; corresponding step sizes 5, 10, 20 seconds, and maximum lag 10 seconds. Figure S2 shows a pattern highly similar to that observed for movement synchrony, indicating that interpersonal synchrony is robust across variations in WCLC parameter settings. Across all combinations, synchrony consistently exceeded surrogate levels, with the 60-second window yielding slightly stronger and more stable effects compared to shorter window sizes.

**Figure S2.**

*Results of t-tests Comparing Observed vs. Surrogate EDA IS*

**3. Observed versus Pseudosychrony**

After parameter sweeping and window selection, we tested observed versus pseudosychrony for all modalities and facets in both conditions, as described in the manuscript. Descriptive statistics for these measures are reported in Table S2 below. Test statistics are reported in the main text.

| **Table S2** | | | | | |
| --- | --- | --- | --- | --- | --- |
| *Descriptive Statistics for Observed and Surrogate IS across modalities, facets, and conditions* | | | | | |
| **Modality** | **Facet** | **solution-focused**  *M (SD)* | | **emotion-focused**  *M (SD)* | |
|  |  | **Observed** | **Surrogate** | **Observed** | **Surrogate** |
| movement | global strength | 0.16 (0.05) | 0.11 (0.02) | 0.15 (0.05) | 0.11 (0.02) |
|  | Maximum strength | 0.17 (0.05) | 0.13 (0.02) | 0.16 (0.04) | 0.12 (0.02) |
|  | global flexibility | 0.01 (0.01) | 0.01 (0.00) | 0.01 (0.01) | 0.01 (0.00) |
|  | peak flexibility | 0.09 (0.02) | 0.08 (0.01) | 0.09 (0.03) | 0.08 (0.01) |
|  | dominance | 0.35 (0.95) | 0.46 (0.10) | 0.29 (1.26) | 0.48 (0.11) |
|  | switching | 4.51 (0.25) | 4.46 (0.08) | 4.45 (0.30) | 4.44 (0.10) |
| HRV | global strength | 0.31 (0.03) | 0.30 (0.01) | 0.31 (0.04) | 0.30 (0.01) |
|  | Maximum strength | 0.49 (0.04) | 0.47 (0.01) | 0.49 (0.04) | 0.47 (0.01) |
|  | global flexibility | 0.05 (0.01) | 0.05 (0.00) | 0.05 (0.01) | 0.05 (0.00) |
|  | peak flexibility | 0.17 (0.02) | 0.16 (0.00) | 0.17 (0.02) | 0.16 (0.01) |
|  | dominance | 0.25 (1.02) | 0.01 (0.12) | 0.15 (1.00) | -0.01 (0.11) |
|  | switching | 4.75 (0.49) | 4.75 (0.07) | 4.72 (0.51) | 4.74 (0.08) |
| EDA_Slope_ | global strength | 0.20 (0.02) | 0.18 (0.01) | 0.20 (0.02) | 0.18 (0.01) |
|  | Maximum strength | 0.43 (0.05) | 0.40 (0.02) | 0.43 (0.04) | 0.40 (0.01) |
|  | global flexibility | 0.03 (0.01) | 0.02 (0.00) | 0.03 (0.01) | 0.02 (0.00) |
|  | peak flexibility | 0.14 (0.04) | 0.12 (0.01) | 0.14 (0.03) | 0.12 (0.01) |
|  | dominance | -0.20 (1.36) | 0.02 (0.13) | 0.19 (1.36) | -0.00 (0.13) |
|  | switching | 5.89 (0.69) | 6.13 (0.08) | 6.01 (0.67) | 6.13 (0.09) |
| EDA Average | global strength | 0.37 (0.06) | 0.32 (0.02) | 0.38 (0.06) | 0.32 (0.02) |
|  | Maximum strength | 0.55 (0.05) | 0.51 (0.02) | 0.56 (0.05) | 0.51 (0.02) |
|  | global flexibility | 0.08 (0.02) | 0.06 (0.01) | 0.08 (0.02) | 0.06 (0.01) |
|  | peak flexibility | 0.18 (0.03) | 0.16 (0.01) | 0.18 (0.02) | 0.16 (0.01) |
|  | dominance | 0.01 (1.16) | 0.01 (0.12) | -0.06 (1.17) | 0.00 (0.15) |
|  | switching | 6.33 (0.58) | 6.51 (0.11) | 6.40 (0.67) | 6.54 (0.10) |
|  | | | | | |

*Note*. Values represent means (M), with Standard Deviation in brackets (SD). Observed = real synchrony (original data). Surrogate = phase-randomized surrogate synchrony.

**4. Condition Differences in Interpersonal Synchrony**

Analyses of condition differences in IS between conditions showed a significant Condition x sequence interaction for both movement global strength and movement peak Strength (as reported in the manuscript). As patterns were similar for global and peak strength, and due to space limitations, the manuscript includes a Figure only for movement global strength. A figure showing the interaction for peak strength is included below.

**Figure S3**

*movement (peak strength) by Condition and sequence.*

*Note.* Colored points represent dyads, with grey lines connecting repeated observations. Half-violins depict the distribution of values, and boxplots summarize the median and interquartile range. White points and black lines indicate condition means. **p* < .001.

**5. Relations between Interpersonal Synchrony and Working alliance**

The tables below report the full results from all linear mixed-effects models examining the association between interpersonal synchrony and working alliance (WAI) outcomes. For each modality and facet, models included both main effects of IS (and condition) and interaction effects between IS and condition (emotion-focused vs. solution-focused), allowing us to test whether IS-WA associations differed across relational contexts. Main effects of condition are not included here, as they were not relevant to this research question. Results of all facets are grouped in one table per modality. Reported statistics include standardized coefficients (β), standard errors (SE), test statistics (*t*), raw *p*-values, and false discovery rate (FDR) corrected *p*-values. Table S3 shows the results for movement IS, Table S4 for HF-HRV IS, Table S5 for EDA Slope, and Table S6 for EDA Average.

| **Table S3.**  *Fixed Effects for Models Predicting WAI from Condition x IS: movement IS* | | | | |
| --- | --- | --- | --- | --- |
|  | | | | |
| **Facet** | **Fixed Effect** | ***β (SE)*** | ***t, p*** | ***P (*_FDR)_** |
| **WAI Collaboration -Client** | | | | |
| global flexibility | Condition x IS | 0.1 (0.07) | t = 1.47, p = .145 | .385 |
| global flexibility | Condition | 0.02 (0.07) | t = 0.31, p = .756 | .851 |
| global strength | Condition interaction | 0.04 (0.06) | t = 0.74, p = .459 | .988 |
| global strength | Main effect | 0.08 (0.08) | t = 1.02, p = .309 | .823 |
| Mean peak Correlation | Condition interaction | 0.04 (0.06) | t = 0.66, p = .510 | .988 |
| Mean peak Correlation | Main effect | 0.08 (0.08) | t = 1.03, p = .306 | .823 |
| Mean peak Lag | Condition interaction | 0.01 (0.07) | t = 0.1, p = .922 | .958 |
| Mean peak Lag | Main effect | -0.04 (0.07) | t = -0.54, p = .589 | .786 |
| SD peak Correlation | Condition interaction | 0.08 (0.06) | t = 1.27, p = .204 | .409 |
| SD peak Correlation | Main effect | 0.02 (0.07) | t = 0.27, p = .785 | .851 |
| SD peak Lag | Condition interaction | 0.04 (0.07) | t = 0.6, p = .550 | .880 |
| SD peak Lag | Main effect | -0.02 (0.07) | t = -0.33, p = .743 | .850 |
| WAI Collaboration - Therapist | | | | |
| global flexibility | Condition interaction | -0.03 (0.07) | t = -0.47, p = .639 | .730 |
| global flexibility | Main effect | 0.07 (0.07) | t = 0.98, p = .331 | .662 |
| global strength | Condition interaction | 0 (0.06) | t = 0.06, p = .954 | .954 |
| global strength | Main effect | 0.11 (0.07) | t = 1.42, p = .158 | .304 |
| Mean peak Correlation | Condition interaction | 0.01 (0.06) | t = 0.14, p = .888 | .954 |
| Mean peak Correlation | Main effect | 0.1 (0.07) | t = 1.33, p = .187 | .304 |
| Mean peak Lag | Condition interaction | -0.02 (0.07) | t = -0.29, p = .772 | .828 |
| Mean peak Lag | Main effect | -0.07 (0.07) | t = -1.03, p = .306 | .873 |
| SD peak Correlation | Condition interaction | -0.07 (0.07) | t = -1, p = .319 | .730 |
| SD peak Correlation | Main effect | 0.08 (0.07) | t = 1.1, p = .273 | .662 |
| SD peak Lag | Condition interaction | 0.06 (0.07) | t = 0.84, p = .399 | .799 |
| SD peak Lag | Main effect | -0.06 (0.07) | t = -0.88, p = .378 | .873 |
| WAI Bond - Client | | | | |
| global flexibility | Condition interaction | 0.07 (0.05) | t = 1.31, p = .193 | .386 |
| global flexibility | Main effect | 0.01 (0.06) | t = 0.26, p = .798 | .798 |
| global strength | Condition interaction | 0.07 (0.04) | t = 1.62, p = .110 | .239 |
| global strength | Main effect | -0.03 (0.07) | t = -0.38, p = .705 | .881 |
| Mean peak Correlation | Condition interaction | 0.07 (0.04) | t = 1.57, p = .119 | .239 |
| Mean peak Correlation | Main effect | -0.02 (0.07) | t = -0.29, p = .771 | .881 |
| Mean peak Lag | Condition interaction | -0.02 (0.05) | t = -0.48, p = .629 | .839 |
| Mean peak Lag | Main effect | -0.13 (0.06) | t = -2.29, p = .024 | .190 |
| SD peak Correlation | Condition interaction | 0.06 (0.05) | t = 1.15, p = .253 | .405 |
| SD peak Correlation | Main effect | 0.03 (0.06) | t = 0.57, p = .570 | .760 |
| SD peak Lag | Condition interaction | -0.01 (0.05) | t = -0.19, p = .851 | .927 |
| SD peak Lag | Main effect | 0.06 (0.06) | t = 1.12, p = .264 | .765 |
| bond WAI - Therapist | | | | |
| global flexibility | Condition interaction | 0.06 (0.05) | t = 1.39, p = .168 | .352 |
| global flexibility | Main effect | 0.05 (0.05) | t = 0.91, p = .363 | .691 |
| global strength | Condition interaction | 0.1 (0.04) | t = 2.57, **p = .012** | **.047** |
| global strength | Main effect | 0.03 (0.06) | t = 0.54, p = .593 | .930 |
| Mean peak Correlation | Condition interaction | 0.1 (0.04) | t = 2.59, **p = .011** | **.047** |
| Mean peak Correlation | Main effect | 0.04 (0.06) | t = 0.63, p = .530 | .930 |
| Mean peak Lag | Condition interaction | -0.06 (0.04) | t = -1.38, p = .171 | .671 |
| Mean peak Lag | Main effect | -0.07 (0.05) | t = -1.41, p = .161 | .912 |
| SD peak Correlation | Condition interaction | 0.06 (0.04) | t = 1.36, p = .176 | .352 |
| SD peak Correlation | Main effect | 0.06 (0.05) | t = 1.27, p = .205 | .691 |
| SD peak Lag | Condition interaction | 0.05 (0.04) | t = 1.12, p = .266 | .671 |
| SD peak Lag | Main effect | 0.02 (0.05) | t = 0.41, p = .684 | .912 |

| **Table S4.**  *Fixed Effects for Models Predicting WAI from Condition x IS: HF-HRV IS* | | | | |
| --- | --- | --- | --- | --- |
| **Facet** | **Effect** | ***β (SE)*** | ***t, p*** | ***p*_FDR_** |
| **Collaboration WAI - Client** | | | | |
| **global flexibility** | Condition interaction | 0.03 (0.07) | t = 0.47, p = .636 | .677 |
| **global flexibility** | Main effect | 0.03 (0.07) | t = 0.4, p = .690 | .851 |
| **global strength** | Condition interaction | 0.01 (0.07) | t = 0.15, p = .884 | .988 |
| **global strength** | Main effect | 0 (0.07) | t = 0.06, p = .948 | .948 |
| **Mean peak Correlation** | Condition interaction | -0.02 (0.07) | t = -0.26, p = .792 | .988 |
| **Mean peak Correlation** | Main effect | -0.01 (0.07) | t = -0.15, p = .882 | .948 |
| **Mean peak Lag** | Condition interaction | -0.06 (0.07) | t = -0.82, p = .414 | .827 |
| **Mean peak Lag** | Main effect | -0.04 (0.07) | t = -0.55, p = .586 | .786 |
| **SD peak Correlation** | Condition interaction | 0.12 (0.07) | t = 1.69, p = .093 | .385 |
| **SD peak Correlation** | Main effect | 0.05 (0.07) | t = 0.74, p = .459 | .851 |
| **SD peak Lag** | Condition interaction | -0.02 (0.07) | t = -0.3, p = .767 | .958 |
| **SD peak Lag** | Main effect | 0.01 (0.07) | t = 0.14, p = .886 | .886 |
| **Collaboration WAI - Therapist** | | | | |
| **global flexibility** | Condition interaction | 0.06 (0.07) | t = 0.9, p = .367 | .730 |
| **global flexibility** | Main effect | 0.09 (0.07) | t = 1.35, p = .178 | .662 |
| **global strength** | Condition interaction | 0.03 (0.07) | t = 0.42, p = .671 | .954 |
| **global strength** | Main effect | 0.11 (0.07) | t = 1.64, p = .103 | .304 |
| **Mean peak Correlation** | Condition interaction | 0.08 (0.07) | t = 1.16, p = .250 | .667 |
| **Mean peak Correlation** | Main effect | 0.09 (0.07) | t = 1.32, p = .189 | .304 |
| **Mean peak Lag** | Condition interaction | 0.01 (0.07) | t = 0.22, p = .824 | .828 |
| **Mean peak Lag** | Main effect | -0.02 (0.07) | t = -0.3, p = .765 | .971 |
| **SD peak Correlation** | Condition interaction | 0.05 (0.07) | t = 0.68, p = .495 | .730 |
| **SD peak Correlation** | Main effect | 0.05 (0.07) | t = 0.79, p = .431 | .689 |
| **SD peak Lag** | Condition interaction | 0.04 (0.07) | t = 0.65, p = .517 | .827 |
| **SD peak Lag** | Main effect | 0 (0.07) | t = 0.04, p = .971 | .971 |
| **bond WAI - Client** | | | | |
| **global flexibility** | Condition interaction | 0.02 (0.06) | t = 0.35, p = .727 | .831 |
| **global flexibility** | Main effect | -0.03 (0.05) | t = -0.66, p = .509 | .760 |
| **global strength** | Condition interaction | 0 (0.05) | t = -0.08, p = .934 | .949 |
| **global strength** | Main effect | -0.04 (0.05) | t = -0.75, p = .457 | .863 |
| **Mean peak Correlation** | Condition interaction | 0 (0.05) | t = 0.06, p = .949 | .949 |
| **Mean peak Correlation** | Main effect | -0.08 (0.05) | t = -1.51, p = .133 | .355 |
| **Mean peak Lag** | Condition interaction | 0 (0.05) | t = -0.09, p = .927 | .927 |
| **Mean peak Lag** | Main effect | -0.05 (0.05) | t = -0.88, p = .382 | .765 |
| **SD peak Correlation** | Condition interaction | 0.05 (0.05) | t = 0.98, p = .329 | .439 |
| **SD peak Correlation** | Main effect | -0.01 (0.05) | t = -0.26, p = .794 | .798 |
| **SD peak Lag** | Condition interaction | -0.04 (0.05) | t = -0.78, p = .436 | .839 |
| **SD peak Lag** | Main effect | 0.01 (0.06) | t = 0.14, p = .887 | .887 |
| **bond WAI - Therapist** | | | | |
| **global flexibility** | Condition interaction | -0.03 (0.05) | t = -0.71, p = .481 | .481 |
| **global flexibility** | Main effect | 0 (0.04) | t = 0.01, p = .995 | .995 |
| **global strength** | Condition interaction | -0.04 (0.05) | t = -0.8, p = .427 | .682 |
| **global strength** | Main effect | 0.02 (0.05) | t = 0.4, p = .690 | .930 |
| **Mean peak Correlation** | Condition interaction | 0.01 (0.04) | t = 0.15, p = .884 | .884 |
| **Mean peak Correlation** | Main effect | 0 (0.05) | t = 0.09, p = .930 | .930 |
| **Mean peak Lag** | Condition interaction | 0.02 (0.04) | t = 0.48, p = .633 | .844 |
| **Mean peak Lag** | Main effect | -0.04 (0.05) | t = -0.94, p = .348 | .912 |
| **SD peak Correlation** | Condition interaction | -0.05 (0.05) | t = -0.97, p = .334 | .445 |
| **SD peak Correlation** | Main effect | 0 (0.04) | t = 0.11, p = .913 | .995 |
| **SD peak Lag** | Condition interaction | 0.04 (0.04) | t = 0.81, p = .419 | .671 |
| **SD peak Lag** | Main effect | 0 (0.05) | t = -0.11, p = .912 | .912 |

| **Table S5.**  *Fixed Effects for Models Predicting WAI from Condition x IS: EDA Slope IS* | | | | |
| --- | --- | --- | --- | --- |
| **Facet** | **Effect** | ***β (SE)*** | ***t, p*** | ***p*_FDR_** |
| **Collaboration WAI -Client** | | | | |
| **global flexibility** | Condition interaction | -0.04 (0.07) | t = -0.53, p = .594 | .677 |
| **global flexibility** | Main effect | -0.06 (0.08) | t = -0.72, p = .472 | .851 |
| **global strength** | Condition interaction | 0 (0.07) | t = 0.01, p = .988 | .988 |
| **global strength** | Main effect | -0.03 (0.07) | t = -0.36, p = .722 | .948 |
| **Mean peak Correlation** | Condition interaction | 0.01 (0.07) | t = 0.22, p = .828 | .988 |
| **Mean peak Correlation** | Main effect | -0.07 (0.07) | t = -1.03, p = .303 | .823 |
| **Mean peak Lag** | Condition interaction | 0.12 (0.07) | t = 1.83, p = .070 | .286 |
| **Mean peak Lag** | Main effect | -0.07 (0.07) | t = -1.06, p = .293 | .780 |
| **SD peak Correlation** | Condition interaction | -0.1 (0.07) | t = -1.59, p = .116 | .385 |
| **SD peak Correlation** | Main effect | -0.02 (0.08) | t = -0.3, p = .763 | .851 |
| **SD peak Lag** | Condition interaction | -0.08 (0.07) | t = -1.25, p = .215 | .573 |
| **SD peak Lag** | Main effect | 0.1 (0.07) | t = 1.42, p = .157 | .772 |
| **Collaboration WAI - Therapist** | | | | |
| **global flexibility** | Condition interaction | 0.04 (0.07) | t = 0.63, p = .529 | .730 |
| **global flexibility** | Main effect | 0.01 (0.08) | t = 0.16, p = .875 | .875 |
| **global strength** | Condition interaction | 0.09 (0.07) | t = 1.28, p = .203 | .667 |
| **global strength** | Main effect | 0 (0.07) | t = -0.02, p = .984 | .986 |
| **Mean peak Correlation** | Condition interaction | 0.09 (0.07) | t = 1.29, p = .197 | .667 |
| **Mean peak Correlation** | Main effect | 0 (0.07) | t = 0.02, p = .986 | .986 |
| **Mean peak Lag** | Condition interaction | 0.12 (0.07) | t = 1.75, p = .082 | .426 |
| **Mean peak Lag** | Main effect | 0.04 (0.07) | t = 0.53, p = .599 | .958 |
| **SD peak Correlation** | Condition interaction | -0.04 (0.07) | t = -0.57, p = .571 | .730 |
| **SD peak Correlation** | Main effect | 0.03 (0.07) | t = 0.35, p = .728 | .870 |
| **SD peak Lag** | Condition interaction | -0.01 (0.07) | t = -0.22, p = .828 | .828 |
| **SD peak Lag** | Main effect | -0.06 (0.07) | t = -0.78, p = .436 | .873 |
| **bond WAI - Client** | | | | |
| **global flexibility** | Condition interaction | -0.09 (0.05) | t = -1.64, p = .104 | .386 |
| **global flexibility** | Main effect | 0.09 (0.07) | t = 1.31, p = .193 | .760 |
| **global strength** | Condition interaction | -0.1 (0.05) | t = -2.02, **p = .046** | .239 |
| **global strength** | Main effect | 0.1 (0.06) | t = 1.72, p = .088 | .355 |
| **Mean peak Correlation** | Condition interaction | -0.08 (0.05) | t = -1.64, p = .105 | .239 |
| **Mean peak Correlation** | Main effect | 0.09 (0.06) | t = 1.61, p = .110 | .355 |
| **Mean peak Lag** | Condition interaction | 0.14 (0.05) | t = 2.78, **p = .007** | .053 |
| **Mean peak Lag** | Main effect | -0.04 (0.06) | t = -0.71, p = .481 | .770 |
| **SD peak Correlation** | Condition interaction | 0 (0.05) | t = -0.06, p = .954 | .954 |
| **SD peak Correlation** | Main effect | -0.05 (0.07) | t = -0.7, p = .485 | .760 |
| **SD peak Lag** | Condition interaction | 0.05 (0.05) | t = 1, p = .319 | .839 |
| **SD peak Lag** | Main effect | -0.02 (0.06) | t = -0.39, p = .700 | .800 |
| **bond WAI t - Therapist** | | | | |
| **global flexibility** | Condition interaction | -0.07 (0.05) | t = -1.49, p = .141 | .352 |
| **global flexibility** | Main effect | 0.05 (0.06) | t = 0.88, p = .382 | .691 |
| **global strength** | Condition interaction | -0.04 (0.04) | t = -0.98, p = .331 | .662 |
| **global strength** | Main effect | 0.03 (0.05) | t = 0.7, p = .485 | .930 |
| **Mean peak Correlation** | Condition interaction | -0.05 (0.04) | t = -1.02, p = .310 | .662 |
| **Mean peak Correlation** | Main effect | 0.03 (0.05) | t = 0.62, p = .536 | .930 |
| **Mean peak Lag** | Condition interaction | 0.13 (0.04) | t = 2.84, **p = .005** | .044 |
| **Mean peak Lag** | Main effect | -0.04 (0.05) | t = -0.74, p = .462 | .912 |
| **SD peak Correlation** | Condition interaction | -0.05 (0.04) | t = -1.19, p = .237 | .380 |
| **SD peak Correlation** | Main effect | 0.02 (0.06) | t = 0.33, p = .745 | .993 |
| **SD peak Lag** | Condition interaction | 0.04 (0.04) | t = 0.92, p = .360 | .671 |
| **SD peak Lag** | Main effect | -0.01 (0.05) | t = -0.11, p = .910 | .912 |

| **Table S6.**  *Fixed Effects for Models Predicting WAI from Condition x IS: EDA Average IS* | | | | |
| --- | --- | --- | --- | --- |
| **Facet** | **Effect** | ***β (SE)*** | ***t, p*** | ***p*_FDR_** |
| **Collaboration WAI - Client** | | | | |
| **global flexibility** | Condition interaction | -0.05 (0.06) | t = -0.81, p = .421 | .674 |
| **global flexibility** | Main effect | -0.01 (0.08) | t = -0.19, p = .851 | .851 |
| **global strength** | Condition interaction | -0.03 (0.06) | t = -0.39, p = .696 | .988 |
| **global strength** | Main effect | -0.02 (0.08) | t = -0.3, p = .761 | .948 |
| **Mean peak Correlation** | Condition interaction | -0.03 (0.07) | t = -0.52, p = .602 | .988 |
| **Mean peak Correlation** | Main effect | -0.01 (0.07) | t = -0.11, p = .913 | .948 |
| **Mean peak Lag** | Condition interaction | 0.12 (0.07) | t = 1.82, p = .071 | .286 |
| **Mean peak Lag** | Main effect | 0.04 (0.07) | t = 0.58, p = .566 | .786 |
| **SD peak Correlation** | Condition interaction | -0.03 (0.07) | t = -0.42, p = .677 | .677 |
| **SD peak Correlation** | Main effect | -0.04 (0.07) | t = -0.54, p = .591 | .851 |
| **SD peak Lag** | Condition interaction | 0 (0.07) | t = 0.05, p = .958 | .958 |
| **SD peak Lag** | Main effect | 0.09 (0.07) | t = 1.31, p = .193 | .772 |
| **Collaboration WAI - Therapist** | | | | |
| **global flexibility** | Condition interaction | 0.01 (0.06) | t = 0.22, p = .827 | .827 |
| **global flexibility** | Main effect | -0.02 (0.07) | t = -0.3, p = .761 | .870 |
| **global strength** | Condition interaction | 0.02 (0.06) | t = 0.31, p = .755 | .954 |
| **global strength** | Main effect | -0.09 (0.07) | t = -1.21, p = .228 | .304 |
| **Mean peak Correlation** | Condition interaction | 0.04 (0.07) | t = 0.67, p = .507 | .954 |
| **Mean peak Correlation** | Main effect | -0.09 (0.07) | t = -1.25, p = .215 | .304 |
| **Mean peak Lag** | Condition interaction | 0.1 (0.07) | t = 1.41, p = .160 | .426 |
| **Mean peak Lag** | Main effect | 0.01 (0.07) | t = 0.12, p = .906 | .971 |
| **SD peak Correlation** | Condition interaction | -0.05 (0.07) | t = -0.72, p = .472 | .730 |
| **SD peak Correlation** | Main effect | 0.08 (0.07) | t = 1.08, p = .282 | .662 |
| **SD peak Lag** | Condition interaction | -0.1 (0.07) | t = -1.53, p = .128 | .426 |
| **SD peak Lag** | Main effect | 0.07 (0.07) | t = 1, p = .317 | .873 |
| **bond WAI - Client** | | | | |
| **global flexibility** | Condition interaction | 0.07 (0.05) | t = 1.52, p = .132 | .386 |
| **global flexibility** | Main effect | -0.07 (0.07) | t = -1.05, p = .298 | .760 |
| **global strength** | Condition interaction | 0.07 (0.04) | t = 1.45, p = .150 | .240 |
| **global strength** | Main effect | -0.04 (0.07) | t = -0.62, p = .540 | .863 |
| **Mean peak Correlation** | Condition interaction | 0.06 (0.05) | t = 1.22, p = .227 | .302 |
| **Mean peak Correlation** | Main effect | -0.01 (0.06) | t = -0.14, p = .885 | .885 |
| **Mean peak Lag** | Condition interaction | 0.03 (0.05) | t = 0.53, p = .595 | .839 |
| **Mean peak Lag** | Main effect | 0.05 (0.05) | t = 0.89, p = .378 | .765 |
| **SD peak Correlation** | Condition interaction | 0.07 (0.05) | t = 1.43, p = .155 | .386 |
| **SD peak Correlation** | Main effect | -0.13 (0.06) | t = -2.11, **p = .037** | .293 |
| **SD peak Lag** | Condition interaction | 0.07 (0.05) | t = 1.44, p = .153 | .612 |
| **SD peak Lag** | Main effect | -0.03 (0.06) | t = -0.48, p = .631 | .800 |
| **bond WAI - Therapist** | | | | |
| **global flexibility** | Condition interaction | -0.04 (0.04) | t = -0.85, p = .397 | .454 |
| **global flexibility** | Main effect | 0.04 (0.06) | t = 0.79, p = .432 | .691 |
| **global strength** | Condition interaction | -0.01 (0.04) | t = -0.3, p = .761 | .884 |
| **global strength** | Main effect | -0.02 (0.06) | t = -0.32, p = .752 | .930 |
| **Mean peak Correlation** | Condition interaction | -0.01 (0.04) | t = -0.16, p = .875 | .884 |
| **Mean peak Correlation** | Main effect | -0.01 (0.05) | t = -0.2, p = .839 | .930 |
| **Mean peak Lag** | Condition interaction | 0.01 (0.05) | t = 0.14, p = .890 | .904 |
| **Mean peak Lag** | Main effect | 0.02 (0.05) | t = 0.36, p = .717 | .912 |
| **SD peak Correlation** | Condition interaction | -0.08 (0.04) | t = -1.78, p = .078 | .352 |
| **SD peak Correlation** | Main effect | 0.07 (0.05) | t = 1.35, p = .180 | .691 |
| **SD peak Lag** | Condition interaction | 0 (0.04) | t = 0.12, p = .904 | .904 |
| **SD peak Lag** | Main effect | 0.01 (0.05) | t = 0.18, p = .856 | .912 |

**6. Relations between Interpersonal Synchrony and Client-rated therapist empathy**

As preregistered, in addition to WAI (our primary outcome measure), we examined the relation between IS and client-rated therapist empathy (using the Barret Lennard Questionnaire). Results of this analysis are presented below. For each IS modality and facet, both the main effect of IS and its interaction with condition (emotion-focused vs. solution-focused) are reported, along with standardized estimates, test statistics, and FDR p-values. Model fit indices, including the intraclass correlation coefficient (ICC) and marginal and conditional R², are also provided. None of the models showed a significant relation between IS and empathy (or an interaction with condition) after FDR correction.

**Table S7.**

*Interpersonal Synchrony Predicting Client-Rated Empathy*

| **IS modality and facet** | **Fixed Effect** | **b (SE)** | **95% CI** | **t, p** | **p (FDR)** | **ICC** | **R² marginal** | **R² conditional** |
| --- | --- | --- | --- | --- | --- | --- | --- | --- |
| EDA average dominance | Condition × Predictor | -0.03 (0.5) | [-1.02, 0.96] | -0.06, .953 | .975 | 0.56 | 0.00 | 0.57 |
|  | Main | 0.45 (0.51) | [-0.57, 1.47] | 0.87, .388 | .897 | 0.56 | 0.00 | 0.57 |
| EDA average global flexibility | Condition × Predictor | 29.44 (23.53) | [-17.28, 76.15] | 1.25, .214 | .839 | 0.57 | 0.01 | 0.57 |
|  | Main | -23.04 (31.14) | [-84.55, 38.47] | -0.74, .460 | .897 | 0.57 | 0.01 | 0.57 |
| EDA average global strength | Condition × Predictor | 15.11 (8.4) | [-1.58, 31.81] | 1.8, .075 | .839 | 0.59 | 0.01 | 0.59 |
|  | Main | 0.47 (11.52) | [-22.27, 23.22] | 0.04, .967 | .978 | 0.59 | 0.01 | 0.59 |
| EDA Average  peak flexiblity | Condition × Predictor | 27.7 (23.26) | [-18.42, 73.82] | 1.19, .236 | .839 | 0.55 | 0.04 | 0.57 |
|  | Main | -72.92 (27.06) | [-126.41, -19.44] | -2.7, **.008** | .757 | 0.55 | 0.04 | 0.57 |
| EDA average peak strength | Condition × Predictor | 12.06 (10.86) | [-9.5, 33.62] | 1.11, .270 | .850 | 0.58 | 0.01 | 0.58 |
|  | Main | 6.07 (13.23) | [-20.08, 32.23] | 0.46, .647 | .913 | 0.58 | 0.01 | 0.58 |
| EDA average switching | Condition × Predictor | 0.23 (0.91) | [-1.57, 2.04] | 0.25, .799 | .957 | 0.56 | 0.00 | 0.57 |
|  | Main | -0.61 (1.01) | [-2.6, 1.38] | -0.6, .547 | .902 | 0.56 | 0.00 | 0.57 |
| EDA slope dominance | Condition × Predictor | 0.89 (0.42) | [0.07, 1.72] | 2.14, .**034** | .839 | 0.56 | 0.03 | 0.57 |
|  | Main | -0.74 (0.44) | [-1.61, 0.12] | -1.7, .092 | .839 | 0.56 | 0.03 | 0.57 |
| EDA slope global flexibility | Condition × Predictor | -14.3 (67.52) | [-148.24, 119.64] | -0.21, .833 | .968 | 0.56 | 0.00 | 0.56 |
|  | Main | 21.56 (81.2) | [-138.94, 182.05] | 0.27, .791 | .957 | 0.56 | 0.00 | 0.56 |
| EDA slope global strength | Condition × Predictor | -28.94 (24.05) | [-76.61, 18.73] | -1.2, .232 | .839 | 0.53 | 0.01 | 0.54 |
|  | Main | 36 (25.66) | [-14.77, 86.77] | 1.4, .163 | .839 | 0.53 | 0.01 | 0.54 |
| EDA slope peak flexibility | Condition × Predictor | 18.92 (16.46) | [-13.76, 51.6] | 1.15, .253 | .839 | 0.56 | 0.01 | 0.57 |
|  | Main | -28.37 (21.52) | [-70.89, 14.15] | -1.32, .189 | .839 | 0.56 | 0.01 | 0.57 |
| EDA slope peak strength | Condition × Predictor | -14.67 (12.75) | [-39.93, 10.59] | -1.15, .252 | .839 | 0.55 | 0.01 | 0.56 |
|  | Main | 14.13 (13.61) | [-12.8, 41.06] | 1.04, .301 | .850 | 0.55 | 0.01 | 0.56 |
| EDA slope switching | Condition × Predictor | 0.87 (0.81) | [-0.73, 2.47] | 1.08, .284 | .850 | 0.58 | 0.00 | 0.59 |
|  | Main | 0.07 (0.93) | [-1.77, 1.91] | 0.08, .939 | .975 | 0.58 | 0.00 | 0.59 |
| HF-HRV dominance | Condition × Predictor | 0.27 (0.57) | [-0.86, 1.4] | 0.48, .635 | .913 | 0.55 | 0.00 | 0.55 |
|  | Main | -0.42 (0.6) | [-1.6, 0.76] | -0.71, .481 | .897 | 0.55 | 0.00 | 0.55 |
| HF-HRV global flexibility | Condition × Predictor | 26.58 (51.45) | [-75.2, 128.36] | 0.52, .606 | .913 | 0.56 | 0.00 | 0.56 |
|  | Main | -16.34 (48.24) | [-111.91, 79.22] | -0.34, .735 | .954 | 0.56 | 0.00 | 0.56 |
| HF-HRV global strength | Condition × Predictor | -7.3 (16.54) | [-40.03, 25.44] | -0.44, .660 | .918 | 0.55 | 0.00 | 0.55 |
|  | Main | -7.69 (16.62) | [-40.58, 25.2] | -0.46, .644 | .913 | 0.55 | 0.00 | 0.55 |
| HF-HRV peak flexibility | Condition × Predictor | 37.03 (28.54) | [-19.44, 93.51] | 1.3, .197 | .839 | 0.56 | 0.01 | 0.57 |
|  | Main | -4.78 (26.96) | [-58.18, 48.61] | -0.18, .860 | .968 | 0.56 | 0.01 | 0.57 |
| HF-HRV peak strength | Condition × Predictor | -0.8 (14.18) | [-28.88, 27.28] | -0.06, .955 | .975 | 0.55 | 0.01 | 0.56 |
|  | Main | -17.33 (14.45) | [-45.93, 11.26] | -1.2, .233 | .839 | 0.55 | 0.01 | 0.56 |
| HF-HRV switching | Condition × Predictor | -0.43 (1.12) | [-2.64, 1.79] | -0.38, .704 | .939 | 0.55 | 0.00 | 0.55 |
|  | Main | -0.21 (1.24) | [-2.66, 2.24] | -0.17, .866 | .968 | 0.55 | 0.00 | 0.55 |
| movement dominance | Condition × Predictor | -0.07 (0.51) | [-1.08, 0.95] | -0.13, .897 | .968 | 0.55 | 0.00 | 0.55 |
|  | Main | -0.04 (0.57) | [-1.18, 1.09] | -0.08, .938 | .975 | 0.55 | 0.00 | 0.55 |
| movement global flexibility | Condition × Predictor | 46.14 (78.03) | [-108.44, 200.72] | 0.59, .555 | .902 | 0.53 | 0.01 | 0.53 |
|  | Main | -63.55 (84.44) | [-230.52, 103.42] | -0.75, .453 | .897 | 0.53 | 0.01 | 0.53 |
| movement global strength | Condition × Predictor | 8.47 (10.06) | [-11.51, 28.45] | 0.84, .402 | .897 | 0.55 | 0.00 | 0.55 |
|  | Main | 1.67 (14.88) | [-27.73, 31.06] | 0.11, .911 | .972 | 0.55 | 0.00 | 0.55 |
| movement Local flexibility | Condition × Predictor | 9.8 (20.9) | [-31.63, 51.22] | 0.47, .640 | .913 | 0.54 | 0.00 | 0.55 |
|  | Main | -18.29 (23.69) | [-65.12, 28.54] | -0.77, .441 | .897 | 0.54 | 0.00 | 0.55 |
| movement peak strength | Condition × Predictor | 7.97 (10.92) | [-13.72, 29.66] | 0.73, .467 | .897 | 0.55 | 0.00 | 0.55 |
|  | Main | 3.99 (16.3) | [-28.21, 36.18] | 0.24, .807 | .957 | 0.55 | 0.00 | 0.55 |
| movement switching | Condition × Predictor | 1.12 (2.02) | [-2.88, 5.11] | 0.55, .581 | .913 | 0.57 | 0.01 | 0.57 |
|  | Main | 2.61 (2.18) | [-1.71, 6.92] | 1.2, .234 | .839 | 0.57 | 0.01 | 0.57 |

7. **Packages versions and references**

***Versions***

R version 4.4.1 (2024-06-14)

**Core packages :** lme4 (1.1-35.5)**,** lmerTest (3.1-3)

**Data manipulation and visualization:** dplyr (1.1-4)**,** tidyr (1.3-1)**,** tibble (3.2-1)**,** stringr (1.5-1)**,** readr (2.1-5)**,** purrr (1.0-2)**,** ggplot2 (3.5-1)**,** scales (1.4-0)

**Statistical analysis and reporting:** emmeans (1.10-6)**,** car (3.1-3)**,** carData (3.0-5)**,** effectsize (1.0-0)**,** performance (0.12-4)**,** parameters (0.24-0)**,** report (0.5-9)**,** rempsyc (0.2-0)**,** datawizard (0.13-0)**,** nlme (3.1-165)**,** Matrix (1.7-0)

**Packages References**

Bates, D., Mächler, M., Bolker, B., & Walker, S. (2015). Fitting linear mixed-effects models using lme4. Journal of Statistical Software, 67(1), 1–48. https://doi.org/10.18637/jss.v067.i01

Bates, D., Maechler, M., & Jagan, M. (2024). Matrix: Sparse and dense matrix classes and methods (Version 1.7-0) [R package]. https://CRAN.R-project.org/package=Matrix

Ben-Shachar, M. S., Lüdecke, D., & Makowski, D. (2020). effectsize: Estimation of effect size indices and standardized parameters. Journal of Open Source Software, 5(56), 2815. https://doi.org/10.21105/joss.02815

Fox, J., & Weisberg, S. (2019). An R companion to applied regression (3rd ed.). Sage. https://www.john-fox.ca/Companion/

Fox, J., Weisberg, S., & Price, B. (2022). carData: Companion to applied regression data sets (Version 3.0-5) [R package]. https://CRAN.R-project.org/package=carData

Kuznetsova, A., Brockhoff, P. B., & Christensen, R. H. B. (2017). lmerTest package: Tests in linear mixed effects models. Journal of Statistical Software, 82(13), 1–26. https://doi.org/10.18637/jss.v082.i13

Lenth, R. (2024). emmeans: Estimated marginal means, aka least-squares means (Version 1.10.6) [R package]. https://CRAN.R-project.org/package=emmeans

Lüdecke, D., Ben-Shachar, M., Patil, I., & Makowski, D. (2020). Extracting, computing and exploring the parameters of statistical models using R. Journal of Open Source Software, 5(53), 2445. https://doi.org/10.21105/joss.02445

Lüdecke, D., Ben-Shachar, M., Patil, I., Waggoner, P., & Makowski, D. (2021). performance: An R package for assessment, comparison and testing of statistical models. Journal of Open Source Software, 6(60), 3139. https://doi.org/10.21105/joss.03139

Makowski, D., Lüdecke, D., Patil, I., Thériault, R., Ben-Shachar, M., & Wiernik, B. (2023). report: Automated results reporting as a practical tool to improve reproducibility and methodological best practices adoption [R package]. https://easystats.github.io/report/

Müller, K., & Wickham, H. (2023). tibble: Simple data frames (Version 3.2.1) [R package]. https://CRAN.R-project.org/package=tibble

Patil, I., Makowski, D., Ben-Shachar, M., Wiernik, B., Bacher, E., & Lüdecke, D. (2022). datawizard: An R package for easy data preparation and statistical transformations. Journal of Open Source Software, 7(78), 4684. https://doi.org/10.21105/joss.04684

Pinheiro, J. C., & Bates, D. M. (2000). Mixed-effects models in S and S-PLUS. Springer. https://doi.org/10.1007/b98882

Pinheiro, J., Bates, D., & R Core Team. (2024). nlme: Linear and nonlinear mixed effects models (Version 3.1-165) [R package]. https://CRAN.R-project.org/package=nlme

R Core Team. (2024). R: A language and environment for statistical computing. R Foundation for Statistical Computing. https://www.R-project.org/

Sklyar, O., Murdoch, D., Smith, M., Eddelbuettel, D., François, R., Soetaert, K., & Ranke, J. (2021). inline: Functions to inline C, C++, Fortran function calls from R (Version 0.3.19) [R package]. https://CRAN.R-project.org/package=inline

Thériault, R. (2023). rempsyc: Convenience functions for psychology. Journal of Open Source Software, 8(87), 5466. https://doi.org/10.21105/joss.05466

Wickham, H. (2016). ggplot2: Elegant graphics for data analysis. Springer.

Wickham, H. (2023). stringr: Simple, consistent wrappers for common string operations (Version 1.5.1) [R package]. https://CRAN.R-project.org/package=stringr

Wickham, H., François, R., Henry, L., Müller, K., & Vaughan, D. (2023). dplyr: A grammar of data manipulation (Version 1.1.4) [R package]. https://CRAN.R-project.org/package=dplyr

Wickham, H., Henry, L. (2023). purrr: Functional programming tools (Version 1.0.2) [R package]. https://CRAN.R-project.org/package=purrr

Wickham, H., Hester, J., & Bryan, J. (2024). readr: Read rectangular text data (Version 2.1.5) [R package]. https://CRAN.R-project.org/package=readr

Wickham, H., Pedersen, T. L., & Seidel, D. (2025). scales: Scale functions for visualization (Version 1.4.0) [R package]. https://CRAN.R-project.org/package=scales

Wickham, H., Vaughan, D., & Girlich, M. (2024). tidyr: Tidy messy data (Version 1.3.1) [R package]. https://CRAN.R-project.org/package=tidyr
